# DAXX promotes centromeric stability independently of ATRX by preventing the accumulation of R-loop-induced DNA double-stranded breaks

**DOI:** 10.1093/nar/gkad1141

**Published:** 2023-12-01

**Authors:** Lia M Pinto, Alexandros Pailas, Max Bondarchenko, Abhishek Bharadwaj Sharma, Katrin Neumann, Anthony J Rizzo, Céline Jeanty, Nathalie Nicot, Carine Racca, Mindy K Graham, Catherine Naughton, Yaqun Liu, Chun-Long Chen, Paul J Meakin, Nick Gilbert, Sébastien Britton, Alan K Meeker, Christopher M Heaphy, Florence Larminat, Eric Van Dyck

**Affiliations:** DNA Repair and Chemoresistance Group, Department of Cancer Research, Luxembourg Institute of Health (LIH), L-1210 Luxembourg, Luxembourg; Faculty of Science, Technology and Communication, University of Luxembourg, L-4365 Esch-sur-Alzette, Luxembourg; Discovery & Translational Science Department, Leeds Institute of Cardiovascular and Metabolic Medicine, University of Leeds, Leeds LS2 9JT, UK; DNA Repair and Chemoresistance Group, Department of Cancer Research, Luxembourg Institute of Health (LIH), L-1210 Luxembourg, Luxembourg; Faculty of Science, Technology and Communication, University of Luxembourg, L-4365 Esch-sur-Alzette, Luxembourg; DNA Repair and Chemoresistance Group, Department of Cancer Research, Luxembourg Institute of Health (LIH), L-1210 Luxembourg, Luxembourg; Faculty of Science, Technology and Communication, University of Luxembourg, L-4365 Esch-sur-Alzette, Luxembourg; DNA Repair and Chemoresistance Group, Department of Cancer Research, Luxembourg Institute of Health (LIH), L-1210 Luxembourg, Luxembourg; DNA Repair and Chemoresistance Group, Department of Cancer Research, Luxembourg Institute of Health (LIH), L-1210 Luxembourg, Luxembourg; Department of Pathology, Johns Hopkins University School of Medicine, Baltimore, MD 21231, USA; DNA Repair and Chemoresistance Group, Department of Cancer Research, Luxembourg Institute of Health (LIH), L-1210 Luxembourg, Luxembourg; Translational Medicine Operations Hub, Luxembourg Institute of Health (LIH), Luxembourg, Luxembourg; Institut de Pharmacologie et Biologie Structurale (IPBS), Université de Toulouse, CNRS, Université Toulouse III – Paul Sabatier (UT3), 31077 Toulouse Cedex 4, France; Department of Pathology, Johns Hopkins University School of Medicine, Baltimore, MD 21231, USA; Medical Research Council Human Genetics Unit, Institute of Genetics and Molecular Medicine, The University of Edinburgh, Edinburgh EH4 1QY, UK; Institut Curie, PSL Research University, CNRS UMR3244, Dynamics of Genetic Information, Sorbonne Université, 75248 Paris Cedex 05, France; Institut Curie, PSL Research University, CNRS UMR3244, Dynamics of Genetic Information, Sorbonne Université, 75248 Paris Cedex 05, France; Discovery & Translational Science Department, Leeds Institute of Cardiovascular and Metabolic Medicine, University of Leeds, Leeds LS2 9JT, UK; Medical Research Council Human Genetics Unit, Institute of Genetics and Molecular Medicine, The University of Edinburgh, Edinburgh EH4 1QY, UK; Institut de Pharmacologie et Biologie Structurale (IPBS), Université de Toulouse, CNRS, Université Toulouse III – Paul Sabatier (UT3), 31077 Toulouse Cedex 4, France; Department of Pathology, Johns Hopkins University School of Medicine, Baltimore, MD 21231, USA; Department of Medicine, Boston University School of Medicine, Boston, MA 02118, USA; Institut de Pharmacologie et Biologie Structurale (IPBS), Université de Toulouse, CNRS, Université Toulouse III – Paul Sabatier (UT3), 31077 Toulouse Cedex 4, France; DNA Repair and Chemoresistance Group, Department of Cancer Research, Luxembourg Institute of Health (LIH), L-1210 Luxembourg, Luxembourg

## Abstract

Maintaining chromatin integrity at the repetitive non-coding DNA sequences underlying centromeres is crucial to prevent replicative stress, DNA breaks and genomic instability. The concerted action of transcriptional repressors, chromatin remodelling complexes and epigenetic factors controls transcription and chromatin structure in these regions. The histone chaperone complex ATRX/DAXX is involved in the establishment and maintenance of centromeric chromatin through the deposition of the histone variant H3.3. ATRX and DAXX have also evolved mutually-independent functions in transcription and chromatin dynamics. Here, using paediatric glioma and pancreatic neuroendocrine tumor cell lines, we identify a novel ATRX-independent function for DAXX in promoting genome stability by preventing transcription-associated R-loop accumulation and DNA double-strand break formation at centromeres. This function of DAXX required its interaction with histone H3.3 but was independent of H3.3 deposition and did not reflect a role in the repression of centromeric transcription. DAXX depletion mobilized BRCA1 at centromeres, in line with BRCA1 role in counteracting centromeric R-loop accumulation. Our results provide novel insights into the mechanisms protecting the human genome from chromosomal instability, as well as potential perspectives in the treatment of cancers with DAXX alterations.

## Graphical Abstract

**Graphical Abstract:**
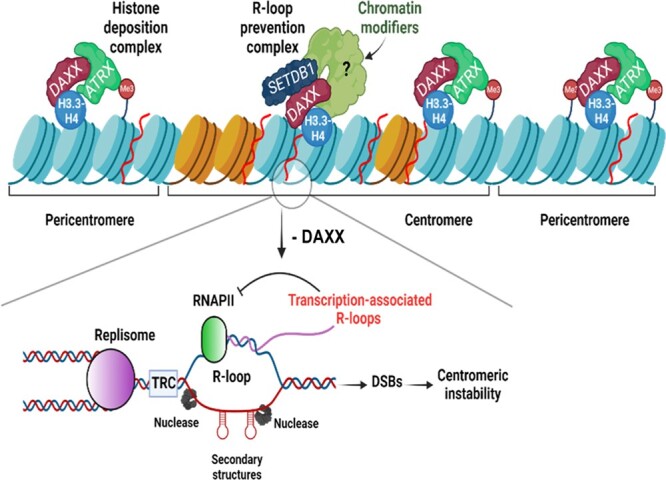

## Introduction

Maintaining chromatin integrity is of paramount importance to prevent chromosomal instability (CIN) (1) – a driving force in cancer - at specific regions of our chromosomes, including centromeres, pericentromeres, telomeres and transposable elements such as endogenous retroviral elements (ERVs). One important feature of these regions is the presence of repetitive DNA sequences. Human centromeres, which are epigenetically defined by the histone H3 variant CENP-A, contain tandem repeats of α-satellite (α-SAT) (171 bp in size) organized in higher-order repeat (HOR) units that can be reiterated in multi-megabase arrays (2–4). Likewise, pericentromeres form multi-megabase regions composed mainly of tandem repeats of human satellite 1–3 (HSat1-3) sequences (4,5), while the telomeric DNA sequence is composed of TTAGGG repeats between 9–15 kb in size (4). Importantly, telomeres and (peri)centromeres are transcribed by RNA polymerase II (RNAPII) into non-coding RNAs (ncRNAs) that participate in the establishment and maintenance of (hetero)chromatin in these regions (2,6,7). A substantial portion of the G-rich TERRA (telomeric repeat-containing RNA) transcripts remains associated with telomeres where they form an integral part of the telomeric heterochromatin structure (8,9), serving in part as a scaffold for the recruitment of heterochromatin factors. On the other hand, careful control of centromere transcription is required for proper CENP-A deposition, CENP-C stabilization and kinetochore function (2,10,11). Notably, excessive accumulation of centromeric ncRNAs disrupts the centrochromatin structure, leading to defects in chromosome segregation and sister-chromatid cohesion (12).

Repetitive sequences can generate complex DNA topology and secondary structures that hamper transcription and replication, including stem-loops, G-quadruplexes (G4) and three-stranded structures called R-loops, which consist of a DNA:RNA hybrid and a displaced single-stranded DNA (ssDNA) strand (13,14). R-loops exert crucial physiological roles, for instance at transcriptional termination sites (TTSs) where they promote fork stalling to prevent DSBs resulting from head-on collisions between the replication and transcription machineries (15). However, R-loops can also be harmful and their unscheduled accumulation has been associated with fork stalling, transcription-replication conflicts, DSB accumulation and genomic instability (5,16–18). Thus, deregulation of (peri)centromeric satellite DNA transcription or depletion of proteins regulating R-loops formation or processing were found to be associated with the accumulation of R-loops and DNA damage (10,16,19,20). Several factors have been involved in the prevention of R-loop accumulation and their resolution, including DNA topoisomerases I and II, the DNA helicases Pif1, DHX9 and Senataxin (SETX), Fanconi Anemia (FA) proteins, and RNase H (reviewed in (21)). At centromeres, the resolution of DNA secondary structures has been shown to involve the DNA2 nuclease/helicase (22) while the homologous recombination (HR) and Fanconi anemia (FA) factor BRCA1 has been proposed to limit centromeric R-loop-associated instability by recruiting SETX (10).

The deposition of histone variant H3.3 is instrumental to the formation and maintenance of (hetero)chromatin at (peri)centromeres, telomeres and other silenced regions, including sites of ERVs (23,24). A histone chaperone complex composed of alpha-thalassemia/mental retardation syndrome X-linked (ATRX) and death domain-associated protein (DAXX) is responsible for H3.3 deposition at these regions (23–28). In addition, centromeric nucleosomes containing H3.3 have been proposed to serve as ‘placeholders’ during the S phase to ensure the subsequent assembly of CENP-A in G1 (29). Through its deposition of H3.3 and the maintenance of a close chromatin state, the ATRX/DAXX complex has also been proposed to afford protection from G-quadruplex-associated replicative stress, including R-loop formation, at telomeres (30) and other regions (31). Notably, loss of ATRX significantly alters the accessibility of repetitive heterochromatin loci, including centromeric regions (32).

In the ATRX/DAXX complex, DAXX provides the H3.3-specific chaperone activity through a central histone-binding domain (HBD) that allows DAXX to wrap around the H3.3-H4 dimer. Another DAXX domain (called the DAXX helical bundle (DHB) or 4 helical bundle (4HB)) mediates DAXX interaction with a centrally-located domain of ATRX. Specific DHB residues critical for this interaction have been described (33). ATRX on its part is a chromatin remodeling protein that contains a C-terminal SWI/SNF2-type ATPase/helicase domain as well as several reader modules of epigenetic marks. Among these is an atypical PHD finger called ATRX-DNMT3-DNMT3L (ADD) domain that functions as a H3-binding module whose binding is promoted by H3K9me3 combined with the absence of H3K4 methylation (34,35). This domain targets DAXX-dependent H3.3 deposition to H3K9me3-enriched heterochromatin.

In addition to forming an H3.3 chaperone complex, ATRX and DAXX have each evolved mutually independent functions in regulation of gene expression, chromatin dynamics and DNA repair (36–40). Recently, DAXX was shown to associate to newly synthesized H3.3-H4 in order to promote, in an ATRX-independent manner, the recruitment of histone methyltransferases mediating H3K9me3 prior to H3.3-H4 deposition onto DNA (41). In addition, while the ATRX/DAXX complex is responsible for the heterochromatinization of regions harbouring ERVs and transposable elements, DAXX was also found to exert an ATRX-independent role in the transcriptional silencing of ERVs (33,42). These observations prompted us to examine whether related ATRX-independent mechanisms involving DAXX also took place to regulate the transcription of repetitive DNA at centromeres and telomeres. In this work, we describe a novel ATRX-independent function for DAXX in preventing transcription-associated R-loop formation at centromeres, as well as DSB accumulation in these regions and centromeric instability.

## Materials and methods

### Cell culture and treatments

The pediatric glioblastoma-derived cell line SF188 and its derivatives were grown in DMEM medium (Gibco) containing 1% ultraglutamine and supplemented with 10% FBS (Gibco), 1% HEPES pH 6.28 (Gibco), 1% sodium pyruvate (Gibco) and 1% penicillin/streptomycin (Lonza), The human pancreatic neuroendocrine tumor cell line BON-1 and its derivatives were grown in DMEM-F12 (Gibco) supplemented with 10% FBS and 1% Pen/Strep. All cell lines were kept at 37°C with 5% CO_2_. For SF188 cells, plasmid selection was achieved using 1 μg/ml puromycin and/or 300 μg/ml G418. Plasmid selection in BON-1 cells was achieved using 400 μg/ml G418. Aphidicolin (MedChem Express, dissolved in DMSO) and colcemid (Millipore, dissolved in water) were used as described in the metaphase spread analysis section. α-amanitin (Sigma-Aldrich, stock solution 1mg/ml in water) was used at a final concentration of 2 μM (DRIP analyses) or 2.5 μM (IF analyses) for 16 h. Nocodazole (Selleckchem, 10 mM in DMSO) was used at a final concentration of 150 ng/ml (500 nM) for 18h.

### Plasmid construction, RNAi and ectopic gene expression

shRNA-mediated depletion of DAXX was achieved using pLKO-Puro-based lentiviral vectors (43). The following pair of shRNA oligonucleotides was used to target the 3′UTR of DAXX: forward primer (Fw) 5′- CTAGGAAGGGATGGACTAAGCTAATCTCGAGATTAGCTTAGTCCATCCCTTCTTTTTG; reverse primer (Rv) 5′-AATTCAAAAAGAAGGGATGGACTAAGCTAATCTCGAGATTAGCTTAGTCCATCCCTTC). The pLKO-Puro-based vector targeting the 3′-UTR of KAP1 (TRCN0000017998) was purchased from Merck. A lentiviral vector expressing non-silencing shRNAs in pLKO (44) was used as negative control.

The siRNA-mediated depletion of SETDB1 and KAP1 was achieved using SMARTpool siGENOME SETDB1 and KAP1 siRNAs (Dharmacon), with siGENOME Non-targeting control siRNA pool I used as a non-silencing siRNA control (Dharmacon). The siRNA-mediated depletion of BRCA1 was achieved using the following siBRCA1 pool: CAACAUGCCCACAGAUCAA, CCAAAGCGAGCAAGAGAAU, UGAUAAAGCUCCAGCAGGA, GAAGGAGCUUUCAUCAUUC (Eurofins Genomics). siRNA transfections were carried out using INTERFERin transfection reagent (Polyplus), according to the manufacturer's instructions.

The vector for ectopic expression of DAXX was constructed by cloning an EcoRI-SalI (blunt) fragment containing DAXX from vector HA-Daxx/pRK5 (45) into the lentiviral vector pCDH-EF1α-MCS-IRES-Neo (System Biosciences) pre-digested with EcoRI-BamHI (blunt). HA-Daxx/pRK5 was a gift from Xiaolu Yang (Addgene plasmid # 52023; http://n2t.net/addgene:52023;RRID:Addgene_52023). The DAXX (Y124A) and DAXX (R371W) mutations were generated by site directed mutagenesis of wild-type DAXX constructs, using the QuickChange Site directed mutagenesis kit (Stratagene) with the following oligonucleotide pairs: (DAXX (Y124A): Fw: 5′-CGGCCAGCCAAGCTCGCTGTCTACATCAATGAGCTCTGC, Rv: 5′- GCAGAGCTCATTGATGTAGACAGCGAGCTTGGCTGGCCG); (DAXX (R371W): Fw: 5′- ACCGGAGTTTGGCCATGAGTTGGCTGGATGAGGTCATCTCC, Rv: 5′- GGAGATGACCTCATCCAGCCAACTCATGGCCAAACTCCGGT). The nucleotide changes were verified by sequencing.

The plasmid expressing GFP-RNase H1 was a kind gift from R. Crouch (NIH, Bethesda, MD, USA) and the control GFP-nuc was purchased from Invitrogen. Plasmids transfections were carried out using JetOptimus (Polyplus) following the manufacturer's instructions. pLenti-hRNase1 was constructed by cloning an EcoR1-Pme1 insert containing hRNaseH1 into pCDH-EF1α-MCS-IRES-Neo.

### ATRX genome editing in SF188 cells

We used the ATRX SF188 knockout clone 1, previously characterized (46), which was generated using two guide RNAs targeting exon 9 of ATRX upstream of all functional domains (the ADD domain, DAXX binding domain and ATPase/helicase domain). This strategy implies that frameshift mutations result in the loss of these critical domains, in addition to potential effects on RNA and protein stability and subcellular localization of ATRX. No wild-type ATRX sequences were observed in this clone, and the following mutations were reported: 15 bp del, 11 bp del and 16 bp del. Using antibodies recognizing distinct epitopes, the authors observed complete loss of nuclear staining of ATRX by IHC, as well as a dramatic loss of ATRX protein by immunoblotting. Notably, the anti-ATRX antibody used in the present study detects a faint band with an apparent electrophoretic mobility similar to that of ATRX (Supplementary Figure S1D). This protein could result from mechanisms such as alternative splicing or internal ribosomal entry site-mediated expression (47) and would therefore also carry small truncations. It could also correspond to very low levels of the 15 bp allele, which would give a protein with a 5 aa deletion. This band represents at most 8–10% of the ATRX levels observed in the parental and the SF188_shCTL cell lines, indicating that this ATRX-KO clone is a relevant model for our study.

### DAXX and ATRX genome editing in BON-1 cells

For DAXX knockout, two CRISPR Cas9 nickase guide RNAs (gRNA-1: 5′-CATGAGGCTCAGAGGAGCTA, gRNA-2: 5′-AGAGGAAGCAGTAGTTCGGG) were designed to target exon 2 of DAXX using MIT CRISPR tool (48). The gRNAs were cloned into the GFP-expressing Cas9n plasmid, PX461, a gift from Feng Zhang (Addgene #48140). Lipofectamine 3000 (Thermo Fisher Scientific) was used to transfect either empty vector PX461 or co-transfect both DAXX gRNA1-PX461 and DAXX gRNA 2-PX461 into BON-1 cells. GFP positive cells were sorted by FACS and cells were plated in 150 mm dishes. Cell colonies were isolated using cloning cylinders (Sigma-Aldrich) and screened for DAXX protein by immunostaining. Promising KO clones were subsequently validated by western blotting and Sanger sequencing.

For ATRX knockout, the same experimental protocol was used, except that the following two guide RNAs targeting exon 9 of ATRX and previously used by Brosnan-Cashman et al. (46) were used: (gRNA-1: 5′-TGGACAACTCCTTTCGACCA and gRNA-2: 5′-TAATGGATGAAAACAACCAA).

### RNA extraction and RT-qPCR

Total RNA was isolated with TRI Reagent (Ambion) using the manufacturer's instructions. Contaminating DNA was removed by treatment with TURBO DNase (Thermo Fisher Scientific), following the manufacturer's instructions. Samples were quantified with a Nanodrop spectrophotometer (Thermo Fisher Scientific) and 1 to 3 μg of RNA were used for reverse transcription and production of cDNA. Reverse transcription was carried out on total RNA with the SuperScript III kit (Thermo Fisher Scientific). Random hexamers (Thermo Fisher Scientific) were used to prepare cDNA for centromeric transcripts analysis. cDNA was diluted to a final concentration of 10–30 ng/μl and used to run qPCR with FAST SYBR reagent (Thermo Fisher Scientific). Relative expression of the transcripts was calculated with the ΔΔCt method. The primer pairs used were: EZRIN (Fw: 5′-TGCCCCACGTCTGAGAATC, Rv: 5′-CGGCGCATATACAACTCATGG), GAPDH (Fw: 5′- AGCCACATCGCTCAGACAC, Rv: 5′- GCCCAATACGACCAAATCC), DAXX (Fw: 5′-AGCTGCTGGCGCTCTATGTG, Rv: 5′- TGCGCTGCTCTATGACACGG), ATRX (Fw: 5′- TTGTACAGCCAGAGCCAGTG, Rv: 5′- GTTGTCCACAAGCAGTGCAG), α-satellite cen1-like (Fw: 5′- TCATTCCCACAAACTGCGTTG, Rv: 5′- TCCAACGAAGGCCACAAGA), D5Z2 (Fw:5′-TTTTTGTGCAATTGGCAAATGGAG, Rv:5′-AGACTGTTTCCTCACTGCTCT).

### Western Blotting (WB) analysis

Cells were harvested, washed once with ice-cold PBS and incubated in 1 x RIPA buffer (Millipore, 20-188) supplemented with protease inhibitor cocktail (Roche, 11697498001) and phosphatase inhibitor cocktail (Roche, 04906837001) for 30 min at 4°C, with occasional vortexing (10 s, three times). Following centrifugation (16 000 g, 15 min) at 4°C, the lysates were stored at -80°C. Protein extracts were quantified using the BRADFORD-solution (Bio-RAD, 5000006) and heated for 10 min at 95°C in LDS-sample loading buffer (Thermo Fisher Scientific, NP0008) containing 50 mM dithiothreitol (Amersham Biosciences, ref. 17-1318-02) before being subjected to SDS-PAGE gel electrophoresis (NuPage™ 4–12% Bis–Tris Gel Invitrogen, NP0322box) and wet transfer to a PVDF membrane (Thermo Fisher Scientific). The membrane was blocked in TBS containing 0.1% TritonX-100 (TBST) and 5% dry milk for 1 h at room temperature (RT) and incubated overnight with the appropriate primary antibody. Following three washes with TBST, the membrane was then incubated with the appropriate horseradish peroxidase (HRP)-conjugated secondary antibody in TBST containing 5% dry milk for 1 h at RT. Following 3 washes with TBST, protein bands were detected with the ECL reagent (Thermo Fisher Scientific) according to the manufacturer instructions and visualized by exposing the membrane to an autoradiography film or to the ImageQuant LAS 4000 acquisition system (GE Healthcare). When necessary, ImageJ software was used to calculate the relative abundance of proteins in the samples.

### Immunofluorescence (IF) analysis

Cells were seeded on coverslips in 24-well plates. Cells were subjected to pre-extraction using cytoskeleton (CSK) buffer (10 mM HEPES–NaOH, 100 mM NaCl, 3 mM MgCl_2_, 300 mM sucrose, pH 7.4) containing 0.5% Triton X-100 as described (49), followed by two washes with cold PBS. Cells were then fixed with 4% PFA (Sigma-Aldrich) at room temperature for 10 min, and washed twice with cold PBS again. To prepare the samples for IF, the cells were treated with a 0.5% solution of Triton X-100 (Carl Roth) in PBS for 10 min, followed by a blocking step with 10% FBS, 0.1% BSA 0.3% Triton X-100 in PBS for 30 min at room temperature. The cells were subsequently incubated overnight at 4°C with primary antibodies diluted in 10% FBS, 0.1% BSA 0.3% Triton X-100. Following three washes with PBS (5 min each), the cells were incubated with fluorophore-conjugated secondary antibody diluted 1:500 in 10% FBS, 0.1% BSA 0.3% Triton X-100. Coverslips were washed again with PBS (3 × 5 min) each and counter-stained with DAPI before being mounted on 1 mm thick glass slides with Fluoromount mounting medium (Sigma-Aldrich). Images were acquired using a confocal microscope (Zeiss LSM880 confocal microscope) with a magnification of 63X or with a confocal laser microscope (FV1000 Olympus) with a Plan-Apochromat 40 × 0.95 lens. The sequential mode was used to acquire images without cross-talk. When comparing experimental conditions, images were taken using the same exposure conditions. Images were then analysed with the ImageJ software. The resulting data was statistically analysed with GraphPad Prism 9.

### Protein extracts and co-immunoprecipitations

Co-IPs for the detection of DAXX-ATRX interactions were carried out using the Pierce HA-Tag Magnetic IP/Co-IP Kit (ThermoScientific, 88838X) according to the manufacturer's instructions, except that ethidium bromide was added to prevent the detection of DNA-dependent protein associations (50). Briefly, cells (∼ 4 million in 15-cm plates) were collected by scraping in cold PBS and centrifugation. Cells lysates were prepared by resuspending the cell pellets in 800 μl IP Lysis/Wash buffer supplemented with protease and phosphatase inhibitors and incubation on ice for 30 min at 4°C, with vortexing (10 s) every 10 min. Extracts were collected following centrifugation at 15 000 rpm for 15 m. For co-IPs, extracts (500 μg of proteins) were brought to 500 μl with IP Lysis/Wash buffer, and ethidium bromide was added to a final concentration of 50 μg/ml. The extracts were incubated on ice for 30 m, followed by centrifugation at 15 000 rpm for 5 min. The cleared extracts were incubated with 0.25 μg (25 μl) of pre-washed anti-HA magnetic beads in a 1.5 ml Eppendorf tube on a spinning wheel for 4 h at 4°C. The beads were then collected using a magnetic stand and washed three times with 300 μl of IP Lysis/Wash buffer containing 50 μg/ml ethidium bromide, followed by a final wash with 300 μl of ethidium bromide-containing IP Lysis/Wash buffer supplemented with NaCl to a final concentration of 200 mM. Elution of the bound proteins was achieved by addition of 100 ml 1X NuPAGE sample buffer (Thermo Fisher Scientific, NP0007), brief vortexing and incubation at 95°C for 10 m in a heating block, followed by separation on a NuPAGE 4–12% Bis–Tris gel (Invitrogen) and immunoblotting.

Co-IPs for the detection of DAXX-histone interactions were carried out as described above, except that the proteins were analysed following separation on a 16% acrylamide:bis-acrylamide (29:1) gel.

### Metaphase spread and fluorescent *in situ* hybridization (FISH)

Metaphase spreads were prepared by adapting the protocol from (51). Briefly, cells were grown to 70% of confluence, then blocked in metaphase with 2–5 μg/ml colcemid (Millipore) for 30–60 min at 37°C. Cells were then resuspended in 5 ml of hypotonic solution (75 mM KCl) and incubated at room temperature for 10 min, followed by fixation with fixing solution (3:1 methanol: acetic acid) three times. Cells were finally dropped on 1 mm thick slides to allow the cells to burst and release the condensed chromosomes (metaphase spread). Metaphase spreads were then let to dry at room temperature in the dark for 2 days, before proceeding to hybridization with FISH probes. Alternatively, samples were stored in fixing solution at −20°C, until use. Metaphase spreads on slides were treated with RNase A (Qiagen) at a concentration of 100 μg/ml for 1 h at 37°C. The hybridization mix was prepared as follows: 50% deionized formamide (Ambion), 10% 20× SSC, 1% TritonX-100 (Carl Roth), 20% Dextran Sulfate (Sigma-Aldrich), 19% H_2_O. For each slide, 100 μl of hybridization mix was used, with the addition of 5 nM PNA C-Rich probes (Eurogentec): Cy3-Telo for telomeres or Cy5-Cent for centromeres. After the RNase A treatment, slides were then quickly washed in 2× SSC and dehydrated for two min in 70%–90%–100% ethanol series, then let to air dry. Slides were warmed up at 70°C for 5 min, before being denatured in 70% formamide 2× SSC pH 7.5 at 70°C for 1.5 min, then quickly transferred to 70% ethanol on ice for 2 min. Slides were completely dehydrated in 90% and 100% ethanol (2 min each) and let to air dry. In the meantime, the probes in hybridization buffer were denatured at 70°C for 5 min and placed back on ice until further use. Finally, they were added to the denatured slides, sealed with a coverslip, and let hybridize for 2 hs in a wet dark box. The coverslip was then gently removed with 2× SSC and slides washed 4 times for 3 min with 2× SSC at 45°C and 4 times for 3 min with 0.1× SSC at 60°C. Slides were finally incubated for 5 min in 4× SSC 0.1% Triton X-100 with DAPI, let dry and coverslips mounted with Fluoromount.

### DNA:RNA immunoprecipitation (DRIP)

DRIP was carried out following the protocol published by Racca *et al.* (10). Briefly, genomic DNA was extracted from 5 million cells using a genomic DNA purification kit (Macherey-Nagel) according to the manufacturer's protocol. DNA was resuspended in 200 μl of elution buffer (5 mM Tris–HCl pH 8) and then fragmented on ice by sonication using a microtip for 3 × 15 s (Branson Sonifier 250) to yield an average fragment size of 800–300 bp. Chromatin fragment size was monitored by agarose gel electrophoresis. Half of each sample was treated with E. coli RNase HI (New England Biolabs) overnight at 37 °C in 1× RNase H reaction buffer. The other half was mock-incubated. After setting aside 1% for input DNA, 2 μg of DNA was used for immunoprecipitation with 400 μl binding buffer (10 mM NaPO4 pH 7.0, 140 mM NaCl, 0.05% Triton X-100) and 5 μg of the S9.6 antibody (produced in house from HB-8730 hybridoma cell line (ATCC)) on a rotative shaker at 4 °C for at least 4 h. At the end of the incubation, 25 μl of protein A magnetic beads (Diagenode) prewashed 2−3 times with binding buffer were added to the DNA/antibody complex and incubated for at least 4 h at 4 °C on a rotative shaker. After four washes with 1 ml binding buffer at 4°C for 10 min each, the beads were eluted using 100 μl of DNA isolation buffer (Diagenode) containing 1 μl proteinase K (20 mg/ml). They were incubated for 15 min in a thermomixer (Eppendorf) at 55°C at a mixing speed of 1200 rpm, and then for 15 min at 100°C at 1200 rpm.. The eluted DNA was analyzed by qPCR to amplify the centromeric α-SAT cen1-like array as described in (10). The DNA:RNA hybrid enrichment was calculated based on the IP/input ratio.

### Chromatin immunoprecipitation (ChIP) assay

ChIP assays were performed using 1 million cells/IP. Cells were crosslinked in medium containing 1% formaldehyde at room temperature for 10 min with rotation. Formaldehyde was quenched by the addition of glycine (final concentration of 125 mM) for 5 min. Cell lysis, nuclei isolation, and immunoprecipitation (IP) were performed using the iDeal ChIP-qPCR kit following the manufacturer**’**s recommendations (Diagenode). Chromatin fractions were sheared on ice for 10 × 10 s using a microtip (Branson Sonifier 250) to yield a DNA fragment size <1000 bp. Chromatin fragment size was monitored by agarose gel electrophoresis after DNA purification. After dilution of chromatin in ChIP reaction mix complemented with protease inhibitors cocktail, samples were incubated overnight at 4°C with the indicated relevant or control antibodies bound to 30 μl Protein A-coated magnetic beads (Diagenode). Beads were captured using a magnetic rack and sequentially washed before elution and DNA purification. Relative quantitation of target sequences in the input and the IP chromatin was performed by qPCR. The fold enrichment of a protein associated to a specific sequence was calculated with respect to the input DNA (1% of the ChIP fraction) and was compared with a ChIP signal obtained using a control non-relevant IgG.

### Real-time quantitative PCR (qPCR) following DRIP and ChIP

qPCR was performed using the SsoFast EvaGreen Supermix (Bio-Rad) supplemented with 0.3 μM specific primer pairs and a CFX96 cycler (Bio-Rad). Each qPCR reaction was performed in technical duplicate. All experiments included a standard curve for each primer pair used and results were analyzed using Bio-Rad Quantity One analysis software.

### Primary and secondary antibodies used for western blotting

Primary antibodies: Actin (Millipore, MAB1501, 1:10 000), DAXX (Sigma-Aldrich, D7810-2ML, 1:1000), DAXX (Cell Signaling Technology, #4533, 1:1000), ATRX (Abcam, ab97508, 1:1000), GAPDH (Cell Signaling Technology, #5174, 1:7000), RNase H1 (Abcam, ab229078, 1:1000), BRCA1 (Bethyl, A300-000A, 1:1000), GFP (Roche, 11814, 1:1000), Vinculin (Santa Cruz, sc-73614, 1:2000), HSP60 (Sigma-Aldrich, H3524, 1:2000), KAP1 (Abcam, ab10483,1:1000), SETDB1 (Proteintech,11231–1-AP,1:1500), Histone H3.3 (Abcam, ab176840, 1:1000), Histone H4 (Cell Signaling Technology, #13919, 1:1000). Secondary antibodies: HRP rabbit (Jackson Laboratory, Cat. No. 111-035-003, 1:50 000), HRP mouse (Amersham/Sigma, Cat. No. GENA931-1ML, 1:10 000).

### Antibodies used for DRIP and ChIP

Mouse anti-RNA/DNA hybrid S9.6 (purified in house from hybridoma HB-8730, 5 μg/DRIP), H3.3 (Abcam, EPR17899, 2 μg/ChIP), control rabbit IgG (Diagenode, C15410206, 2 μg/ChIP) and control mouse IgG (Diagenode, C15400001-100, 5 μg/DRIP).

### Primary and secondary antibodies used for immunofluorescence

Primary antibodies: γH2A.X (Millipore, Merck 05–636, 1:1000), γH2A.X (Cell Signalling Technology, #9718, 1:1000), human CREST serum (AntibodiesInc, SKU 15–234, 1:800), R-loops (S9.6, Absolute Antibody, Ab01137-23.0, 1:400), TRF2 (Abcam, ab13579, 1:500), BRCA1 (Santa-Cruz Biotech, sc-6954 (D-9), 1:250), 53BP1 (Merck, MAB3802, 1:1000), 53BP1 (Abcam,ab36823, 1:1500), DAXX (Cell Signaling Technology, #4533, 1:25), a-tubulin (Proteintech, 66031-1-Ig, 1:750), ATRX (Abcam, ab97508, 1:500). Secondary antibodies: anti-mouse AlexaFluor 488 (Invitrogen, A11001, 1:500), anti-mouse AlexaFluor 555 (Invitrogen, A21422, 1:500), anti-mouse AlexaFluor 647 (Invitrogen, A-21235, 1:500), anti-rabbit AlexaFluor 488 (Invitrogen, A-11008, 1:500), anti-rabbit AlexaFluor 555 (Invitrogen, A21428, 1:500), anti-rabbit AlexaFluor 647 (Invitrogen, A21244, 1:500), anti-human AlexaFluor 647 (Invitrogen, A-21445, 1:500).

### Statistical analysis

For each experiment 3–5 biological replicates were run, unless specified otherwise. Statistical analysis of the data was done with either GraphPad Prism 9 or earlier versions, as well as R. All datasets were initially cleared of outliers using the ROUT method on GraphPad Prism. Datasets were then tested for normality with the Shapiro-Wilk test to determine whether parametric or non-parametric tests were needed. To compare the means of the distributions between two samples, we used Student t-test (parametric) or Mann-Whitney test (non-parametric). For multiple comparisons, we used one-way ANOVA (parametric) or Kruskal-Wallis test (non-parametric). Statistical significance was defined as: *P*-value > 0.05 ns = not significant; *P*< 0.05 *; *P*< 0.01 **; *P*< 0.001 ***; *P*< 0.0001 ****.

## Results

### An ATRX-independent function for DAXX in maintaining centromeric stability

**Figure 1.**
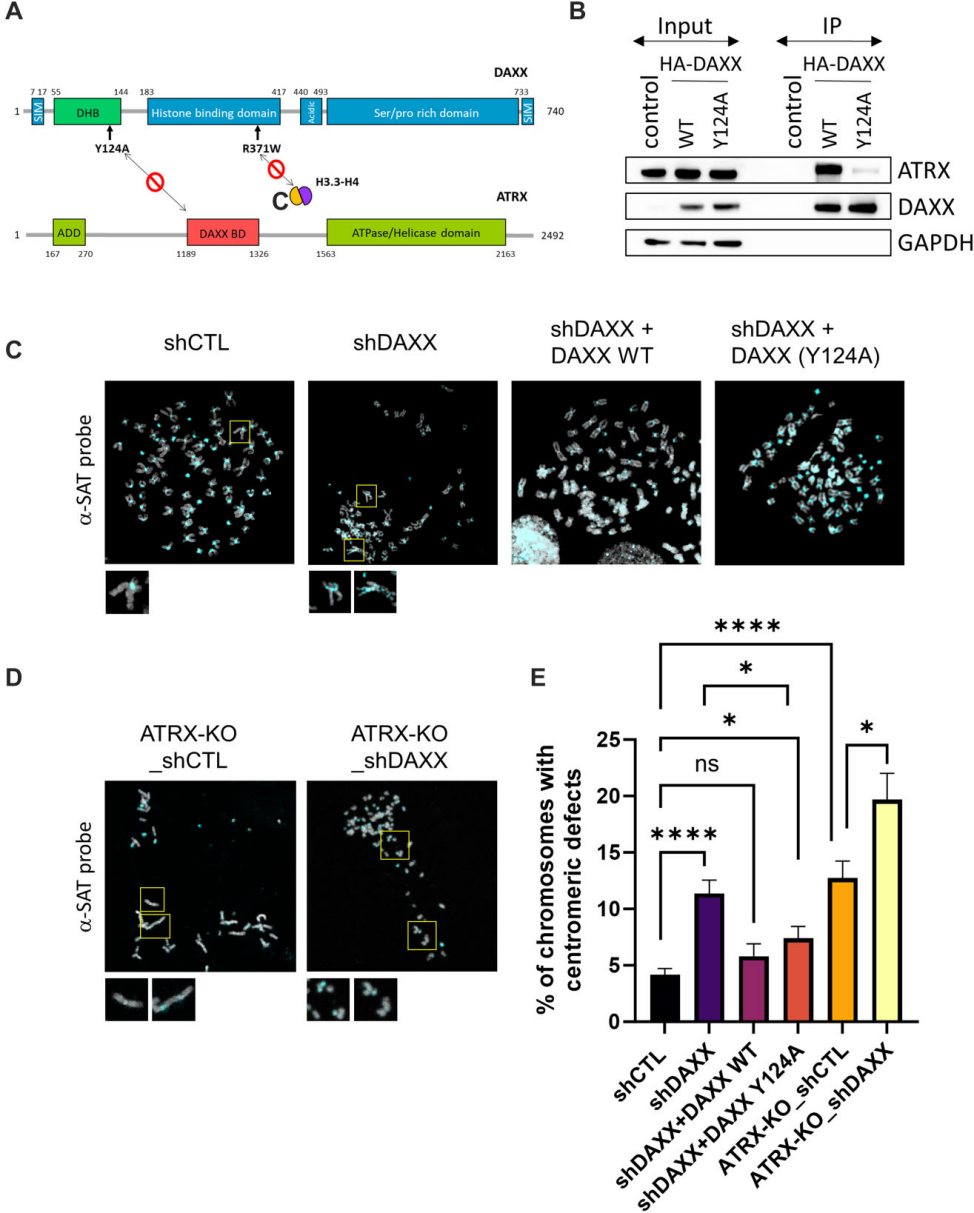
An ATRX-independent function for DAXX in maintaining centromeric stability. (**A**) Scheme representing the important functional domains of the DAXX and ATRX proteins (after references (33,37,38)), as well as the position of the Y124A mutation within the DAXX helical bundle (DHB), which disrupts DAXX-ATRX interaction (33). Also shown is the position of the R371W mutation within the histone binding domain of DAXX, also characterized in this study. (**B**) The DAXX (Y124A) mutation disrupts the interaction with ATRX. Representative western blot analysis of DAXX and ATRX in immunoprecipitates obtained from SF188 cells expressing an empty vector (control) or the indicated HA-tagged DAXX constructs, using an anti-HA antibody. See Supplementary Figure S1C for quantification. (**C, D**) FISH on metaphase spread of the indicated SF188 cell lines with Cy5-CEN (α-SAT) PNA probe (cyan). **(E)** Quantification of centromere defects per metaphase spread in the indicated cell lines. All experiments account for three biological replicates. At least 15 spreads (per biological replicate) for each sample and condition were used for the quantification. Statistical significance is reported as: *P*-value < 0.05 *, *P*-value < 0.0001 ****, *P*-value > 0.05 ns. The error bars represent the standard error of mean (s.e.m.).

Since mutations affecting the H3.3/ATRX/DAXX axis are frequently observed in pediatric high grade glioma (pHGG) (52), we chose the pHGG-derived adherent cell line SF188 (H3.3/ATRX/DAXX WT, p53 mutant) to generate an experimental system for the identification of novel ATRX-independent functions for DAXX. We created otherwise isogenic SF188 cell line derivatives that solely differed in the expression of DAXX, by expressing either a control, non-silencing shRNA (shCTL) or one directed against the 3′-UTR of the DAXX mRNA (shDAXX), to facilitate rescue experiments with ectopic DAXX constructs lacking the 3′-UTR. The DAXX-depleted cells were concomitantly engineered to express either HA-tagged DAXX wild-type (DAXX WT) or HA-tagged DAXX (Y124A), a mutant DAXX protein with a mutation analogous to mouse Y130A, previously shown to be unable to interact with ATRX (33) (Figure 1A). DAXX knock-down, as well as the ectopic expression of DAXX WT and DAXX Y124A were verified at both mRNA and protein levels (Supplementary Figure S1A, B). DAXX depletion or its ectopic expression did not affect the protein levels of ATRX (Supplementary Figure S1B). We also verified by co-immunoprecipitation experiments using anti-HA antibodies, the lack of physical interaction between DAXX (Y124A) and ATRX in our SF188 cells, as predicted from the study of the mouse Y130A mutation (33). These experiments were carried out in the presence of ethidium bromide to prevent the detection of DNA-dependent protein associations (50). Compared with DAXX WT, our data indicate that the DAXX (Y124A) mutation reduced the interaction with ATRX by >95% (Figure 1B and Supplementary Figure S1C), thus validating the use of this mutant in our study. Finally, to complement our DAXX reconstitution system (Figure 1A) and help delineate the role of DAXX and ATRX using otherwise isogenic cells, we also characterized an ATRX-knockout SF188 cell line (ATRX-KO) previously generated by CRISPR (46), as well as a DAXX-depleted derivative thereof (ATRX-KO_shDAXX) (Supplementary Figure S1D).

To uncover roles for DAXX in preserving genomic stability, and given the importance of the ATRX/DAXX histone chaperone complex in the maintenance of chromatin integrity at telomeres and centromeres, we examined metaphase spreads of the SF188 derivatives for telomere and centromere defects, via fluorescent *in situ* hybridization (FISH), using Cy3/5-conjugated PNA probes specific for telomeric TTAGGG repeats and centromeric α-satellite (α-SAT) repeats. To facilitate the detection of even subtle defects, we pre-treated the cells with low concentrations of the DNA polymerase inhibitor aphidicolin (0.4 μM for 24 h) to induce mild replicative stress and highlight fragile sites in the chromatin (51). We found a 3-fold increase (*P*-value < 0.05) in telomeric aberrations (i.e. duplication, single or double loss and ectopic localization of the telomeric signal) in DAXX-depleted cells compared to unsilenced cells (Supplementary Figure S1E, F). Notably, DAXX WT but not DAXX (Y124A) rescued this phenotype (Supplementary Figure S1E, F), indicating that its interaction with ATRX is needed for DAXX-mediated prevention of telomere instability. Consistent with this notion, loss of ATRX also led to a significant ∼3 fold increase in telomeric defects (*P*-value < 0.01), which was not exacerbated significantly by DAXX depletion (Supplementary Figure S1F). Taken together, our data indicate that telomere stability is mediated primarily by the ATRX-DAXX complex, in line with previous reports (27,53,54).

We then scored for loss and/or duplication of the centromeric signal, as well as the presence of a high number of acrocentric chromosomes, and observed a ∼3 fold increase (*P*-value < 0.01) in the percentage of chromosomes bearing centromere defects in DAXX-depleted cells compared to unsilenced cells (Figure 1C–E). Notably, contrasting with our findings at telomeres, the DAXX (Y124A) mutant partly decreased the centromere-defective phenotype when ectopically expressed (Figure 1C and E), suggesting an ATRX-independent function for DAXX in maintaining centromeric stability. Corroborating this notion, loss of ATRX also elicited a significant ∼3-fold increase in centromeric defects (*P*-value < 0.01), with the combined depletion of DAXX and ATRX KO resulting in an additive effect (∼5-fold increase in centromeric defects compared to ATRX-KO alone, *P*-value < 0.01) (Figure 1D, E). Collectively our data suggest that unlike telomere stability, which is mediated primarily by the ATRX-DAXX complex, the stability of centromeres also involves an ATRX-independent activity for DAXX.

### Prevention of spontaneous DNA damage at centromeres requires the ATRX/DAXX complex but also an ATRX-independent DAXX activity

**Figure 2.**
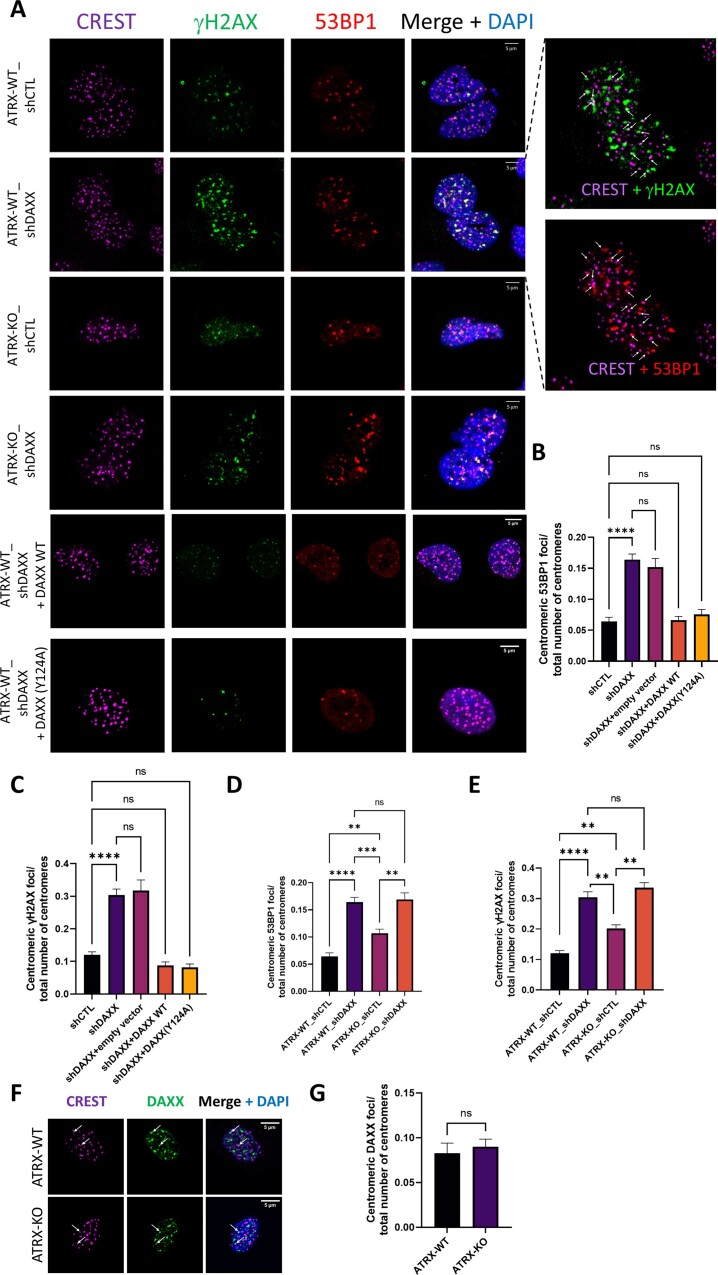
DAXX depletion increases spontaneous DNA damage at centromeres. (**A**) Representative IF images of CREST foci (centromeres, magenta), γH2AX foci (green) and 53BP1 (red) in the indicated SF188 cell derivatives. White arrows in the rightmost panels point to representative CREST-γH2AX and CREST-53BP1 colocalized foci. (**B–E**) Related quantification of the centromeric 53BP1 foci (B and D) or centromeric γH2AX (C and E), reported as fraction of total CREST foci. The experiments account for five biological replicates. At least 80 nuclei for each sample and condition were used for every biological replicate for quantification. Scale bar = 5 μm. Statistical significance is reported as: *P*-value < 0.01 **, *P*-value < 0.001 ***, *P*-value < 0.001 **** *P*-value > 0.05 ns. The error bars represent the s.e.m. (**F–G**) Representative IF images of CREST foci (magenta) and DAXX foci (green) in WT and ATRX-KO cells (F), and related quantification of centromeric DAXX foci (G). Arrows in (F) point to representative colocalized DAXX-CREST foci. The experiment account for three biological replicates. At least 50 nuclei for each sample and condition were used for every biological replicate for quantification. Scale bar = 5 μm. Statistical significance is reported as: *P*-value > 0.05 ns.

We next undertook to address the cause behind the increase in chromosomal instability observed in DAXX-depleted cells. We first examined DSB formation by monitoring 53BP1 foci (55–58) by indirect immunofluorescence (IF) microscopy and observed a significant increase (*P*-value < 0.001) in the overall levels of 53BP1 foci in DAXX-KD cells as compared to control cells (Figure 2A and Supplementary Figure S2A). To rule out the possibility that DAXX knockdown or its ectopic expression could influence the cell cycle and therefore the accumulation of DSBs during replication, we verified with propidium iodide (PI) staining that the distribution of cells across G1, S and G2/M phases was not affected by DAXX status (Supplementary Figure S2B).

We next focused on DSBs at centromeres visualized using antibodies derived from a human CREST patient serum that detects centromeric proteins, including CENP-A and CENP-B. DAXX-depleted cells displayed a significant (*P*-value < 0.001) 2.6-fold increase in increase in 53BP1 foci that localized at centromeres (defined by CREST staining) (Figure 2A, B). Remarkably, the higher numbers of centromeric DSBs reflected an ATRX-independent role for DAXX at centromeres, as revealed by rescue experiments with DAXX WT and DAXX (Y124A) (Figure 2A, B). Similar observations were made when DNA damage was visualized using γH2AX foci, a marker of DSB and replication stress (59–61) (2.5 fold increase in colocalized γH2AX-CREST foci between shCTL and shDAXX, *P*-value < 0.001) (Figure 2A and C). Since DAXX can deposit CENP-A ectopically under certain circumstances (62), we verified that the number of CREST foci and their intensity was not affected by depletion or ectopic expression of DAXX (Supplementary Figure S2C, D). ATRX-KO cells also accumulated centromeric DSBs compared to control cells, but to a smaller extent than DAXX-depleted cells (1.78-fold and 1.6-fold increase in colocalized 53BP1-CREST foci and colocalized γH2AX-CREST foci, respectively; Figure 2A, D and E). In addition, we observed comparable numbers of centromeric foci in DAXX-depleted cells and in ATRX-KO_shDAXX cells (Figure 2A, D and E). Taken together, our observations indicate that DSB prevention at centromeres requires the ATRX/DAXX complex but also a DAXX activity that is independent of ATRX. Our rescue experiments with ectopic constructs also suggest that this ATRX-independent, centromere-protective function of DAXX takes precedence over the ATRX/DAXX complex. Finally, as a step to establish that DAXX plays a direct role in protecting centromeres independently of ATRX, we visualized DAXX and CREST using IF in control and ATRX-KO cells. As shown in Figure 2F and G, the number of centromeric DAXX foci was not affected by ATRX loss, indicating that DAXX can localize to centromere independently of ATRX.

DAXX depletion also led to a significant increase (∼2.5-fold, *P*-value < 0.001) in telomere dysfunction induced foci (TIFs) compared to unsilenced cells, as assessed following visualization of colocalized γH2AX and TRF2 foci (Supplementary Figure S2E, F). A milder increase (∼1.7-fold, *P*-value < 0.01) was observed in ATRX-KO cells (Supplementary Figure S2E and G), which was exacerbated by DAXX depletion. Notably, both ectopic expression of DAXX WT and DAXX (Y124A) reduced the amount of TIFs to control levels (Supplementary Figure S2E, F).

Collectively, these results suggest a novel, ATRX-independent function for DAXX in preventing DSB formation at centromeres and telomeres. Unlike for centromeric DSBs, however, the rescue afforded by the DAXX (Y124A) construct at the TIF level (Supplementary Figure S2E, F) did not translate at the level of genomic stability (Supplementary Figure S1E, F). This observation suggests that although DAXX alone is sufficient to protect centromeres and telomeres from DSB accumulation, telomeres rely on both DAXX and ATRX for their stability, likely in part due to the role of ATRX in the resolution of G4 structures which telomeres are prone to form (53,63).

### DAXX prevents the accumulation of R-loops at centromeres

**Figure 3.**
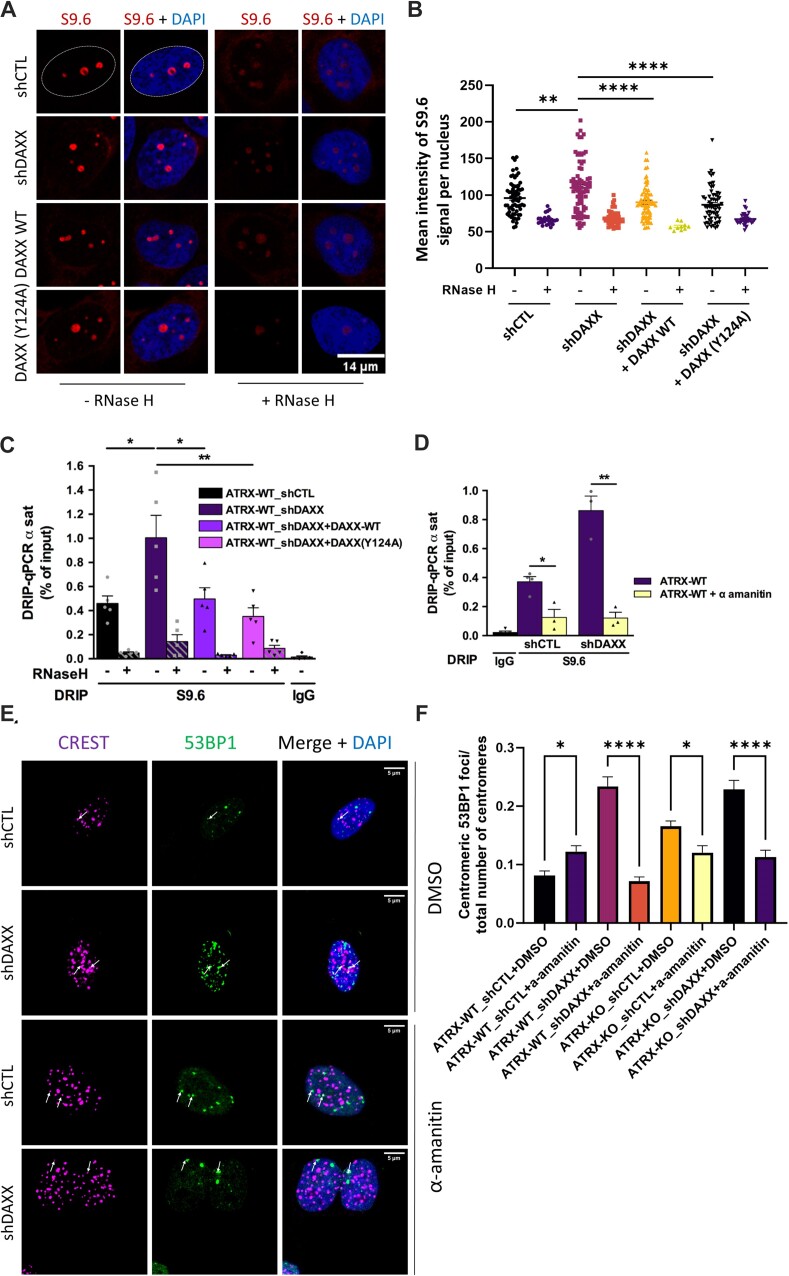
DAXX knockdown elicits centromeric R-loop accumulation. (**A**) Indirect IF analysis with the R-loop-specific antibody S9.6 (red) in the indicated SF188 cell derivatives treated or not with RNase H. The nuclear contour in the upper left panel shows an example of the area that was used for quantification. (**B**) Related quantification of S9.6 mean intensity per cell (with or without RNase H). Error bars represent the s.e.m. (**C**) DRIP-qPCR analysis at α-SAT repeats in DAXX-depleted cells expressing the indicated DAXX constructs. RNase H treatment was performed on half of each sample prior to IP with the S9.6 antibody. Displayed is the mean R-loop enrichment (as percent input) ± s.e.m. The data correspond to five independent experiments. (D–F) Inhibition of transcription by α-amanitin suppresses the accumulation of centromeric R-loops and DSBs elicited by DAXX depletion. (**D**) DRIP-qPCR analysis at α-SAT repeats in WT and DAXX-depleted cells left untreated or treated with α-amanitin as described in Materials and Methods. The data correspond to four independent experiments. (**E**) Representative IF images of 53BP1 foci (green) and CREST foci (magenta) in the indicated ATRX-WT derivative cell lines left untreated or treated with α-amanitin. White arrows point to representative co-localizing 53BP1 and CREST foci. Scale bar: 5 μm. See Supplementary Figure S3B for images of ATRX-KO derivative cell lines. (**F**) Related quantification of centromeric 53BP1 foci in the indicated cell lines left untreated or treated with α-amanitin. Also see Supplementary Figure S3B for representative IF images of ATRX-KO cell derivatives. All IF experiments account for three biological replicates. At least 50 nuclei for each sample and condition were used for every biological replicate for quantification. Scale bar = 5 μm. Statistical significance is reported as: *P*-value < 0.05 *, *P*-value < 0.01 **, *P*-value < 0.0001 ****. The error bars represent the s.e.m.

Given that unscheduled R-loops can generate DSBs at centromeres and pericentromeres (10,64), we next examined R-loop accumulation in DAXX-depleted and ATRX-KO cells. In preliminary experiments, we analysed the intensity of R-loop signals given by the S9.6 antibody, which specifically recognises DNA:RNA hybrids, in control and DAXX-depleted cells. Since this antibody is known to produce a strong background signal (65), we included control coverslips pre-treated with RNase H, which specifically degrades DNA:RNA hybrids, to show that the S9.6 signals obtained were RNase H-sensitive. Compared to control cells, DAXX-depleted cells displayed a significant increase (*P*-value < 0.01) in S9.6 signal intensity, which was restored to control levels when either DAXX WT or DAXX (Y124A) were ectopically expressed (Figure 3A, B). We next investigated specifically the accumulation of R-loops at centromeres using DNA:RNA immunoprecipitation (DRIP) (65) followed by qPCR to amplify α-SAT arrays. As shown in Figure 3C, DAXX depletion elicited a significant increase in centromeric R-loops as compared to control cells (2.19 fold, *P*-value < 0.05). Importantly, expression of either DAXX WT or DAXX (Y124A) rescued the levels of R-loops at centromeres to those of control cells (Figure 3C). As reported for telomeres (30), loss of ATRX also resulted in the accumulation of R-loops at centromeres (Supplementary Figure S3A).

We next confirmed that transcription inhibition by α-amanitin reduced the levels of centromeric R-loops significantly in both control and DAXX-depleted cells (Figure 3D). Importantly, transcription inhibition also reduced significantly the accumulation of centromeric DSBs seen in DAXX-depleted cells, as well as in ATRX-KO and ATRX-KO_shDAXX cells (Figure 3E and F, and Supplementary Figure S3B), linking this accumulation to transcription-associated centromeric R-loops. Noteworthy, unlike in DAXX and ATRX-depleted cells, transcription inhibition by α-amanitin elicited a small increase in centromeric DSB in WT cells (Figure 3F). Taken together, our observations indicate that like DSBs, R-loop prevention at centromeres requires not only ATRX, but also an ATRX-independent DAXX activity. In the remaining part of this work, we focused on the mechanisms by which DAXX fosters its ATRX-independent centromere protection function.

### Overexpression of RNase H reduces R-loop and DSB formation at centromeres and prevents the chromosomal instability associated with loss of DAXX

**Figure 4.**
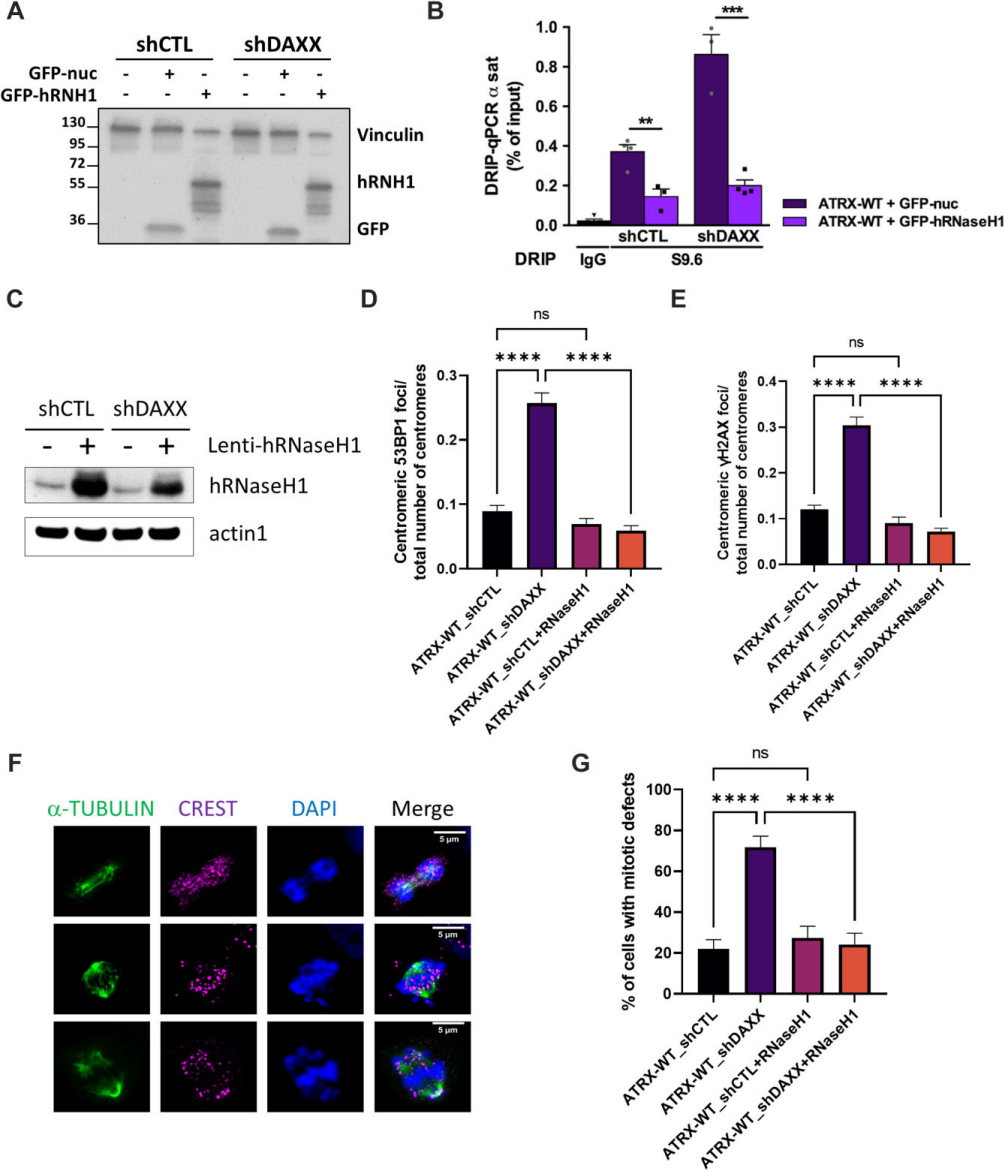
Overexpression of hRNaseH1 suppresses R-loop formation and DSB accumulation at the centromeres of DAXX-depleted cells. (**A**). Immunoblot analysis of GFP-hRNH1 and GFP-nuc expression in whole cell extracts of shCTL and shDAXX SF188 cells. (**B**). DRIP-qPCR analysis at α-SAT repeats in control and DAXX-depleted cells expressing either GFP-hRNH1 or GFP-nuc. RNase H treatment was performed on half of each sample prior to IP with the S9.6 antibody. The graph shows the mean R-loop enrichment (as percent of input) +/- s.e.m., n ≥ 3 independent experiments. (**C**). Immunoblot analysis of hRNAseH1 in whole cell extracts of the indicated cell lines stably transduced with an empty lentiviral vector or a vector expressing hRNAseH1. (**D-E**). Quantification of centromeric 53BP1 foci (**D**) and centromeric γH2AX foci (**E**) in the indicated cell lines expressing or not hRNAseH1. See Supplementary Figure S4A for representative IF images. (**F, G**). Representative IF images of mitotic defects scored in control and DAXX-depleted cells stably expressing or not hRNAseH1 (**F**). Mitotically arrested cells were stained with DAPI, CREST (magenta) and a-tubulin (green). Shown from upper to lower panel are examples of anaphase bridges, misaligned chromosomes, centromeres not associated with chromatin and lagging chromosomes. (**G**) Related quantification showing the mean percentage of cells with mitotic defects from *n* ≥ 60 anaphase/telophase cells analysed for each condition in two biological replicates. The experiments in (D-E) account for three biological replicates. At least 50 nuclei for each sample and condition were used for every biological replicate for quantification. Scale bar = 5 μm. Statistical significance is reported as: *P*-value < 0.01 **, *P*-value < 0.001 ***, *P*-value < 0.0001 ****, *P*-value > 0.05 ns. The error bars represent the s.e.m.

To further establish a causal relationship between the loss of DAXX, the accumulation of transcription-associated R-loop and DSBs at centromeres and centromeric instability, we transiently expressed a GFP-tagged human RNase H1 (hRNaseH1) construct to downregulate the levels of RNA–DNA hybrids in control and DAXX-depleted cells (Figure 4A). Expression of GFP-hRNaseH1 led to a significant reduction in centromeric R-loops, compared to cells transfected with a nucleus-targeting GFP construct (2.7 fold in control cells, *P*< 0.01 and 6.7 fold in shDAXX cells, *P*< 0.005; Figure 4A, B). We next stably overexpressed hRNaseH1 in control and DAXX-depleted cells (Figure 4C) and examined the accumulation of centromeric DSBs by IF in these cells. As shown in Figure 4D (colocalized 53BP1-CREST foci) and 4E (colocalized γH2AX-CREST foci) (see Supplementary Figure S4A for IF images), hRNaseH1 overexpression led to a dramatic reduction in the numbers of centromeric DSBs in DAXX-depleted cells. A similar observation was made in DAXX-depleted cells transiently expressing hRNaseH1-GFP (Supplementary Figure S4B, C). Collectively, these experiments demonstrate that the formation of centromeric DSBs is caused by transcription-associated R-loops accumulating at the centromeres of DAXX-depleted cells.

We next examined the impact of hRNaseH1 overexpression on the CIN phenotype of DAXX-depleted. To this end, we scored mitotic aberrations in DAXX-depleted cells stably expressing or not hRNAseH1, arrested in prometaphase using nocodazole for 16 h, washed and released into drug-free medium for 45 min and fixed after washout. As shown in Figure 4F and G, loss of DAXX led to a 2.4-fold increase (*P*-value < 0.001) in anaphase/telophase cells displaying mitotic aberrations, compared to control cells. Importantly, this phenotype was rescued by overexpression of hRNAseH1 (Figure 4F, G). These observations demonstrate that R-loops accumulation at centromeric chromatin drives the centromeric instability and the mitotic aberrations observed in DAXX-depleted cells.

### DAXX knockdown leads BRCA1 recruitment at centromeres

**Figure 5.**
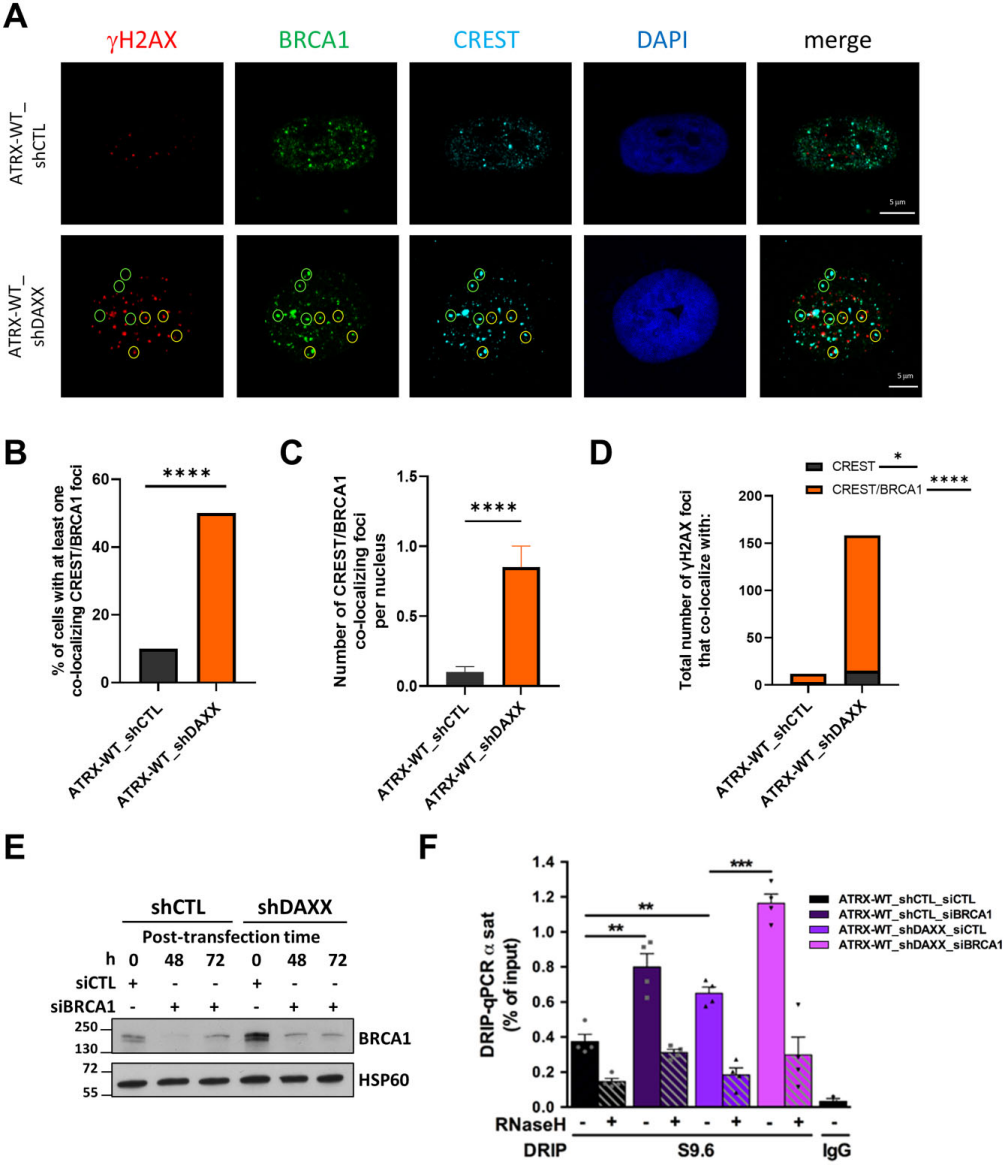
BRCA1 accumulates at centromeres in the absence of DAXX. (A–D) IF images of γH2AX foci (red), BRCA1 foci (green) and CREST foci (cyan) in control and DAXX-depleted SF188 cells. Green circles indicate representative examples of CREST-BRCA1 colocalized foci while yellow circles indicate foci where all 3 markers colocalize or are in close juxtaposition (**A**). (B–D) Related quantification of the number of cells displaying BRCA1 association with undamaged centromeres (**B**), as well as the number of colocalized CREST-BRCA1 foci per nucleus in these cells (**C**). (**D**) Graph showing the number of CREST-γH2AX and CREST-γH2AX-BRCA1 colocalized foci in control and DAXX-depleted cells. At least 50 nuclei for each sample and condition were used for every biological replicate for quantification. Scale bar = 5 μm. Statistical significance is reported as: *P*-value < 0.05 *, *P*-value < 0.01 **, *P*-value < 0.0001 ****. (**E**) Immunoblot of whole-cell lysates showing the efficiency of the siRNA-mediated depletion of BRCA1 in SF188 shCTL and shDAXX cells. HSP60 was used as a loading control. (**F**) DRIP-qPCR analysis at α-SAT repeats in control and DAXX-depleted cells following RNAi against BRCA1. RNase H treatment was performed on half of each sample prior to IP with the S9.6 antibody. The graph shows the mean R-loop enrichment (as percent of input) ± s.e.m., *n* = 4 independent experiments.

R-loops arising at centromeric chromatin have been shown to recruit BRCA1, which then counteracts their accumulation (10). We reasoned that the accumulation of R-loops elicited by DAXX loss should mobilize BRCA1 at centromeres. We therefore examined the presence of BRCA1 foci at centromeres in control and DAXX-depleted cells (Figure 5A–D). When focusing on centromeres devoid of DSBs, we found that loss of DAXX led to a ∼ 4 fold increase (*P*-value < 0.001) in the number of cells with BRCA1 foci at centromeres (Figure 5A, B), with the number of centromeric BRCA1 foci/nucleus raising by about 5-fold in such cells (Figure 5A and C). These observations are consistent with our DRIP data showing that DAXX depletion elicits centromeric R-loop accumulation (Figure 3C).

As observed before (Figure 2A, B), the loss of DAXX led to an increase in the numbers of centromeric DSBs (*P*-value < 0.001) (Figure 5D). Notably, most of these centromeric DSBs were associated with BRCA1, as assessed by the analysis of CREST-γH2AX-BRCA1 colocalized foci (Figure 5A and D). While the recruitment of BRCA1 to damaged centromeres is in line with previous studies showing that BRCA1 contributes to the repair of DSBs arising at centromeres (66), our observations also suggest that the activities orchestrated by BRCA1 to counteract centromeric R-loops are not sufficient to prevent R-loop accumulation and DSBs in the absence of DAXX.

Given the complementary role of DAXX and BRCA1 in preventing and counteracting centromeric R-loop accumulation, respectively, we examined the impact of the double inactivation of these factors. To this end, we investigated centromeric R-loop accumulation in control and DAXX-depleted SF188 cells transfected with siRNAs targeting BRCA1 (Figure 5E). Similar to DAXX depletion, siRNA-mediated BRCA1 depletion in control cells led to strong accumulation of R-loops at centromeres (Figure 5F). Remarkably, the double knockdown of DAXX and BRCA1 resulted in an additive effect, further increasing the centromeric R-loops levels (Figure 5F), highlighting the complementary role of DAXX and BRCA1 for the prevention/resolution of centromeric R-loops.

### Interrelationship between DAXX, KAP1 and SETDB1

ERV transcriptional repression involves a complex of DAXX (stabilized by the H3.3-H4 heterodimer) with the histone methyltransferase SETDB1, the histone deacetylase HDAC1 and the heterochromatin building factor and co-repressor KAP1 (35). Likewise, the ATRX-independent promotion of H3.3K9me3 by DAXX involves the recruitment of the histone methyltransferases SUV39H1 and SETDB1 (41). As a step to identify the factors that collaborate with DAXX to promote centromeric protection in human cells, we tested the epistatic relationships between DAXX, KAP1 and SETDB1 in the prevention of centromeric DSBs. To this end, we examined the impact of RNAi-mediated depletions of these factors (Supplementary Figure S5A) on the accumulation of centromeric DSBs in WT and DAXX-depleted SF188 cells. The single, siRNA-mediated depletion of KAP1 in WT cells had no significant impact on the numbers of colocalized 53BP1-CREST foci (Supplementary Figure S5B, C). This finding was confirmed using shRNA-mediated KAP1-depleted cells (Supplementary Figure S5D, E), and further corroborated by the observation that such cells accumulated levels of R-loops similar to control cells (Supplementary Figure S5F). In contrast, SETDB1 depletion led to a significant increase in centromeric DSBs in WT cells (Supplementary Figure S5B, C). Notably, siRNA-mediated depletion of SETDB1 in KAP1-depleted cells led to levels of centromeric DSBs comparable to that seen in SETDB1-depleted cells alone, while depletion of either KAP1 or SETDB1 in DAXX-depleted cells did not exacerbate the numbers of centromeric DSBs resulting from the single depletion of DAXX (Supplementary Figure S5B, C). Collectively, these experiments suggest that the ATRX-independent function of DAXX in preventing centromeric DSBs involves SETDB1, while KAP1 may have a more limited (or redundant) role.

### The ATRX-independent, centromeric protection activity of DAXX requires its interaction with H3.3 but does not involve H3.3 deposition

**Figure 6.**
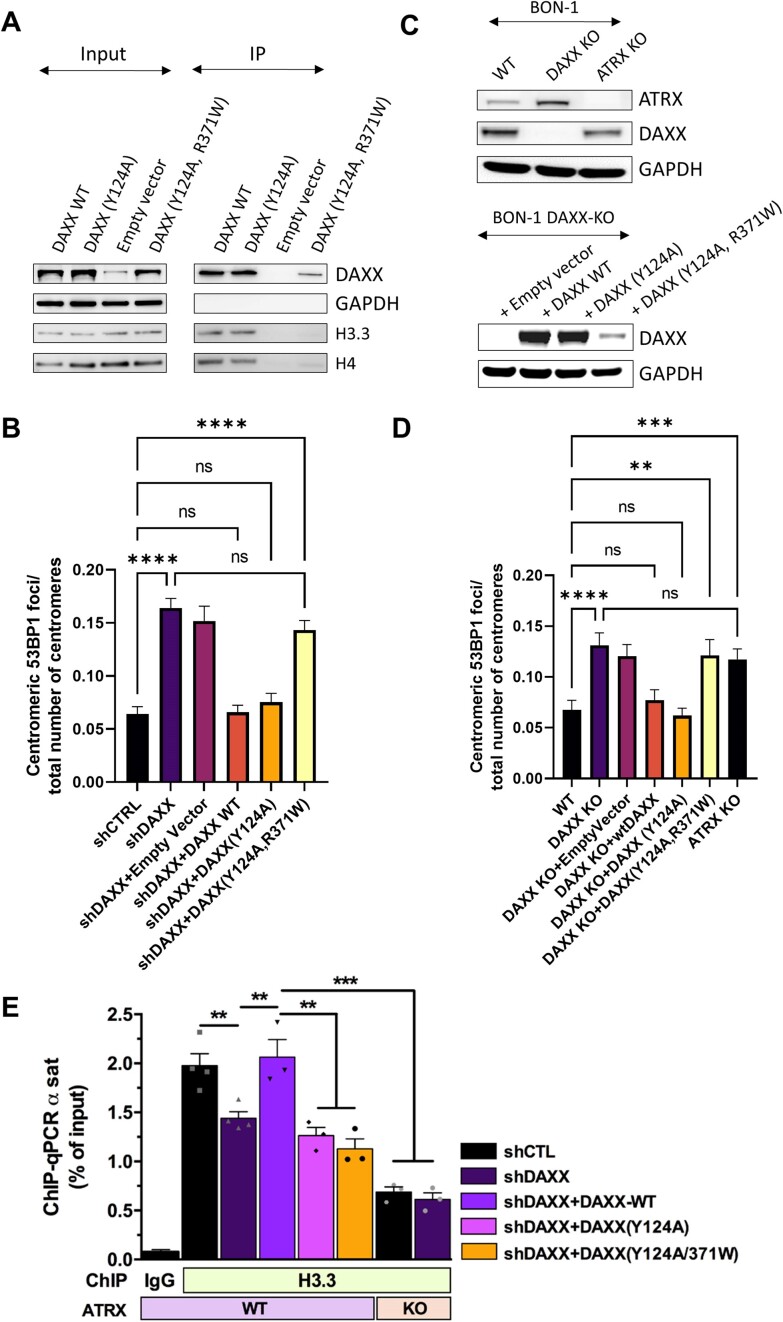
Interplay between DAXX and H3.3 in centromere protection. (**A**). Representative western blot analysis of DAXX, H3.3 and H4 in anti-HA immunoprecipitates from SF188 cells expressing an empty vector (EV) or the indicated HA-tagged DAXX constructs, using an anti-HA antibody. (**B**) Quantification of the CREST foci co-localising with 53BP1 foci in DAXX-depleted cells expressing the indicated DAXX constructs (see Supplementary Figure S6C and Figure 2A for representative IF images). (**C**) Immunoblot analysis verifying the loss of DAXX and ATRX proteins in the BON-1 DAXX-KO and ATRX-KO derivatives, respectively, generated by CRISPR-cas9, as well as the expression of the indicated DAXX constructs. (**D**) Quantification of the CREST foci co-localising with 53BP1 foci in the indicated BON-1 cell derivatives. See Supplementary Figure S6E for representative IF images. *N* = 3. At least 50 nuclei for each sample and condition were used for every biological replicate for quantification. (**E**) ChIP-qPCR analyses showing the relative levels of H3.3 at centromeres (cen1-like primers) in the indicated cell lines, as percent of input. *n* ≥ 3 independent experiments. Scale bar = 5 μm. Statistical significance is reported as: *P*-value < 0.01 **, *P*-value < 0.001 ***, *P*-value < 0,0001 ****, *P*-value > 0.05 ns. The error bars represent the s.e.m.

We next tested whether the interaction with H3.3-H4 was important for DAXX ability to prevent DSB accumulation at centromeres. To this end, we generated a DAXX (Y124A) mutant also harbouring a mutation previously shown to disrupt the interaction with H3.3 and H4 (33). Specifically, we introduced the clinically-relevant R371W mutation, corresponding to the murine R377W mutation, which has been identified in breast ductal carcinoma and acute myeloid leukaemia (source: Catalogue of Somatic Mutations in Cancer (COSMIC), https://cancer.sanger.ac.uk/cosmic) (See Figure 1A for a cartoon depicting the position of the R371W mutation in DAXX). We verified by Co-IP that the R371W mutation reduced DAXX interaction with H3.3 and H4 to almost background levels (Figure 6A and Supplementary Figure S6A). Notably, the protein levels of DAXX (Y124A, R371W) appeared reduced by ∼ 2 fold compared to DAXX WT (Figure 6A and Supplementary Figure S6B), although its mRNA levels were similar to that of DAXX WT (Supplementary Figure S6C), in line with the notion that DAXX is stabilized by its association with H3.3 (33). When taking into account the reduced stability of the DAXX (Y124A, R371W) mutant, we found that its interaction with H3.3 and H4 was decreased by > 80% (Supplementary Figure S6D). We next analysed centromeric DSBs in these cells by IF staining of CREST and 53BP1. Unlike DAXX (Y124A), DAXX (Y124A, R371W) failed to block the accumulation of centromeric DSBs seen in DAXX-depleted cells (Figure 6B and Supplementary Figure S6E).

As a step to generalize our findings and considering the clinical relevance of DAXX and ATRX defects in human pancreatic neuroendocrine tumors (PanNETs) (67,68), we next examined centromeric DSB accumulation in the panNET cell line BON-1, as well as in ATRX-KO and DAXX-KO derivatives thereof, which we generated by CRISPR, and in DAXX-KO BON-1 cells expressing WT DAXX, DAXX (Y124A) or DAXX (Y124A, R371W) (Figure 6C). As observed in SF188 cells, IF analysis of colocalized CREST-53BP1 foci revealed the accumulation of centromeric DSBs upon DAXX loss (Figure 6D and Supplementary Figure S6F). A comparable accumulation was observed in ATRX-KO cells (Figure 6D and Supplementary Figure S6F). Importantly, as observed in SF188 cells, the accumulation of centromeric DSBs seen in DAXX-KO cells could be rescued by ectopic expression of the ATRX-binding mutant DAXX (Y124A) but not by the DAXX (Y124A, R371W) mutant defective for both ATRX and histone binding (Figure 6D and Supplementary Figure S6F). Noteworthy, the protein levels of the DAXX (Y124A, R371W) mutant also appeared reduced compared to DAXX WT (Figure 6C), as observed in SF188 (Figure 6A) and in line with previous findings (33). Collectively, our observations demonstrate that DAXX exerts an ATRX-independent, H3.3-dependent activity to protect centromeres from DNA damage.

Having shown that the binding of DAXX to H3.3–H4 is required for its ATRX-independent activity at centromeres, we next measured the relative abundance of H3.3 at centromeres by ChIP, in WT cells, as well as ATRX-KO and DAXX-depleted SF188 derivatives. Compared to WT cells and DAXX-depleted cells, ATRX knockout elicited the strongest decrease in H3.3 occupancy at centromeres (Figure 6E). Notably, DAXX depletion did not exacerbate the decrease in H3.3 abundance seen in ATRX-KO cells. Remarkably, WT DAXX, but not the ATRX-binding mutant DAXX (Y124A), was able to rescue H3.3 incorporation in DAXX-depleted cells (Figure 6E). These observations indicate that centromeric deposition of H3.3 is mediated largely by ATRX in SF188 cells and that the contribution of DAXX to this process is dependent on its interaction with ATRX. They also suggest that, like for ERVs (33), the ATRX-independent, centromeric protection activity of DAXX, which requires its association with H3.3, does not involve H3.3 deposition.

### Differential impact of DAXX and ATRX loss on the transcription of centromeric DNA repeats

The similarities observed between DAXX activity at centromeres and its ATRX-independent function in the repression of ERVs (33), together with the documented contribution of high transcription on R-loop formation (69,70), prompted us to examine the impact of DAXX depletion on centromeric ncRNA transcription. To this end, we used RT-qPCR to examine the transcription of the repetitive DNA sequences underlying specific centromeric α-SAT arrays from different chromosomes ((cen1-like (chromosome 1, 5, 19) (71,72), D5Z2 (chromosome 5) (73)) in SF188 cells lacking DAXX and/or ATRX.

As illustrated in Supplementary Figure 7A, DAXX depletion led to the significant downregulation of centromeric ncRNAs levels at the examined regions (∼20% (*P*-value < 0.05) and 45% (*P*-value < 0.01) decrease in the regions targeted by cen1-like and D5Z2, respectively). In contrast, loss of ATRX led to a significant ∼3–4 fold increase (*P*-value < 0.0001) in alphoid DNA transcripts in these regions, with a similar upregulation also observed in ATRX-KO_shDAXX cells (Supplementary Figure S7A). Taken together, these observations indicate different roles for DAXX and ATRX in centromeric transcription. In addition they suggest that DAXX depletion cannot mitigate the transcriptional upregulation seen upon ATRX loss, implying a hierarchy in the sequence of events orchestrated by ATRX-DAXX and DAXX itself to modulate transcription at centromeres. Noteworthy, ectopic expression of DAXX, but not the DAXX (Y124A) and DAXX Y124A, R371W) mutants, in DAXX-depleted cells, had a more robust impact on transcription repression than DAXX depletion itself (*P*-value < 0.01) at both regions (Supplementary Figure 7A). These observations suggest that ectopic DAXX can exert a transcriptional repression impact through its recruitment of additional ATRX molecules to centromeres. In support of this notion, we found that DAXX depletion led to a significant, but not complete, decrease (∼30%, *P*-value < 0.05) in ATRX foci in SF188 cells (Supplementary Figure S7B, C), indicating that DAXX plays an important role in the recruitment of ATRX to centromeres.

In summary, our observations suggest that the ATRX-independent function of DAXX in preventing transcription-associated R-loops does not reflect a function in the repression of alphoid DNA transcription.

## Discussion

**Figure 7.**
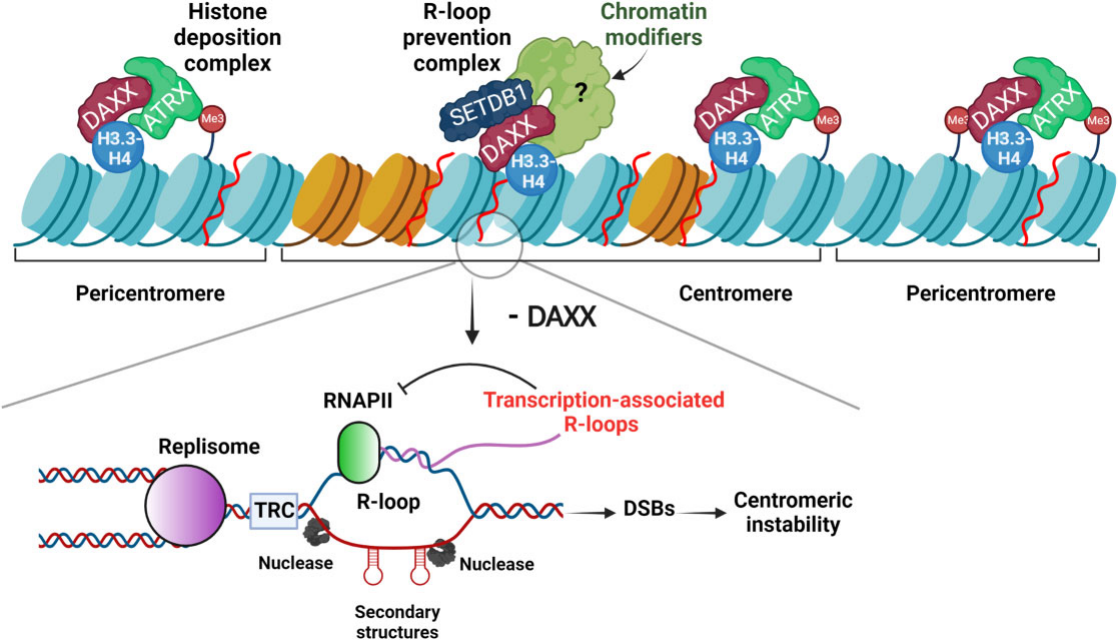
DAXX exerts an ATRX-independent function in the prevention of R-loop-associated centromeric instability - model integrating the present findings. Upper panel: Depicted are pericentromeric regions surrounding a centromeric region characterized by CENP-A containing nucleosomes (orange). In addition to promoting H3.3 deposition at centromeres as part of the ATRX-DAXX complex, DAXX also prevents unscheduled R-loop accumulation. This activity is independent of ATRX, stabilized by DAXX interaction with H3.3-H4 and involves SETDB1 and possibly other chromatin modifiers. Loss of DAXX results in the uncontrolled accumulation of transcription-associated R-loops at centromeres. Although such R-loops favor the recruitment of BRCA1, shown to counteract R-loop accumulation at centromeres (10), our data indicate that the action of BRCA1 is not sufficient in the absence of DAXX, resulting in DSB accumulation and centromeric instability. DSBs associated with unscheduled R-loops occurring at repetitive centromeric DNA sequences could result from transcription-replication conflicts and/or the action of nucleases on the displaced ssDNA, including at harmful structures such as hairpins. We propose that, upon loss of DAXX, the centromeric R-loops produced during transcription inhibit subsequent rounds of transcription (represented by the inhibitory feedback arrow), leading to the observed decrease in centromeric transcripts levels in DAXX-depleted cells.

Centromeres are regarded as a paragon of repetitive fragile sites. The repetitive nature of their satellite DNA has profound effects on centromere topology, DNA replication and recombination, and the aberrant rearrangements often elicited by centromere breaks make an important contribution to the chromosome instability phenotype associated with diseases and cancer (5,74). In this work, we identified a novel, ATRX-independent function for DAXX in fostering centromeric stability. Specifically, we established a causal link between loss of DAXX, transcription-associated R-loop-induced DSBs at centromeres and centromeric instability (Figure 7).

Although R-loops play a physiological role in several processes including DNA repair (66), their unscheduled or excessive formation can lead to DNA damage, hyper-recombination and genomic instability (22,75,76). Thus, while timely-regulated centromeric R-loops have been shown to ensure accurate chromosome segregation (77), our data support a model whereby unscheduled R-loop accumulation elicited by the loss of DAXX result in DNA damage and centromeric instability. DAXX, therefore, identifies a novel mechanism to prevent the formation of unscheduled R-loops and their deleterious effects. It is noteworthy that unscheduled R-loops arising at sites of actively transcribed genes are also associated with a general relaxation of the chromatin (78). Indeed, a similar relaxation is observed at telomeres in ATRX/DAXX-deficient cells displaying impaired H3.3 deposition (54), supporting our findings that DAXX may impact R-loop and DSB formation at centromeres both through its ATRX-independent activity and as part of the ATRX-DAXX H3.3 deposition complex. We found that ATRX loss caused upregulation of centromeric transcription. Thus, the ATRX-DAXX complex may modulate R-loop formation by orchestrating histone H3.3 deposition and heterochromatin accessibility to alter centromeric promoter activity and/or transcriptional elongation (79). This mechanism may be related to the one whereby ATRX protects cells from G-quadruplex DNA-mediated stress (31). Unlike ATRX loss, DAXX depletion elicited the down regulation of transcription at the centromeres we studied. Noteworthy, not only high transcription but also characteristics of the chromatin (69,78) and DNA topology (80) can dictate R-loop formation. Thus, R-loop formation in yeast was strongly increased near polyA tracts which disfavor nucleosome binding (69). Likewise, TDR3-mediated arginine methylation of histone H4 was shown to facilitate the recruitment of topoisomerase IIIB to chromatin to prevent R-loop accumulation at the c-MYC locus (81). Therefore, DAXX may also modulate R-loop formation at satellite DNA sequences independently of H3.3 deposition by affecting histone modifications, as suggested by the involvement of SETDB1 in its centromere-protective function. Noteworthy, R-loop formation can inhibit further transcription by direct mechanisms (e.g. through R-loop-mediated transcription blockage or modulation of RNA polymerase pausing) (70,82–84) or more indirect mechanisms (e.g. through R-loop-induced changes in chromatin structure or DNA topology) (78,85,86). We therefore propose that the centromeric R-loops produced during transcription in DAXX-depleted cells inhibit subsequent rounds of transcription, leading to the observed decrease in centromeric transcripts levels in these cells. Notably, DAXX depletion has recently been shown to cause a decrease in the ncRNA transcript levels of at least certain centromeres (87).

Several parallels can be drawn between the function of DAXX at centromeres and its role in the repression of ERVs. Like for ERVs (33,42), the integrity of centromeres relies both on ATRX/DAXX-mediated H3.3 deposition (24,88) and an ATRX-independent activity exerted by DAXX. In addition, our data suggest that, similar to ERV transcriptional repression (33), prevention of centromeric instability by DAXX (i) requires its stabilization by the H3.3–H4 heterodimer, (ii) occurs independently of H3.3 deposition into chromatin and iii) involves the participation of the histone methyltransferase SETDB1. Unlike for ERV transcriptional repression, however, we found that KAP1 played little role, if any, in the prevention of centromeric DSBs. The identity of the factors that collaborate with DAXX to ensure centromeric protection in human cells warrants further investigation. However, it is likely that the nature and action of these factors are dictated at least in part by specificities of the centrochromatin, which displays unique features such as CENP-A, as well as the presence of both repressive and activating histone marks (2). For instance, although KAP1 was found in association with heterochromatin protein 1 (HP1) in pericentromeric heterochromatin in drosophila (89), its tethering to the centromere of a human artificial chromosome (HAC) caused loss of HAC centromeric transcription and inactivation of its centromere (90), indicating that tight modulation of these factors might be required.

The R-loops that accumulated at centromeres in DAXX-depleted cells recruited BRCA1, in line with previous studies showing the ability of BRCA1 to detect DNA:RNA hybrids (10,91) and antagonize centromeric R-loop accumulation, likely through the recruitment of the helicase SETX (10). In addition to promoting R-loop resolution, BRCA1 has also been shown to be involved in the repair of DSBs arising at centromeres (66). The double knockdown of DAXX and BRCA1 resulted in an additive effect in R-loop accumulation at centromeres. In addition, our observation that a substantial portion of BRCA1 foci colocalized with centromeric DSBs in DAXX-depleted cells also suggest that, in the absence of DAXX, BRCA1 action is not sufficient to counteract centromeric R-loop accumulation.

The evidence indicates that H3.3 deposition still occurs, although at lower levels, in cells depleted of ATRX/DAXX (54) (33). We found that centromeric deposition of H3.3 is mediated largely by ATRX in SF188 cells, with the contribution of DAXX to this process depending on its interaction with ATRX. The more severe impact of ATRX loss on H3.3 centromeric occupancy compared to DAXX loss suggests that histone chaperones with partially-redundant functions (92) can mediate H3.3 deposition at centromeres in DAXX-depleted cells. These may include the H3.1 chaperone CAF1 which can be found associated with H3.3 in DAXX null mouse cells (28), as well as the H3.3 chaperone HIRA, which normally deposits H3.3 in transcribed gene bodies and promoters (26,93) but has previously been proposed to mistarget H3.3 at ectopic sites in the absence of DAXX (88). How such DAXX-independent H3.3 deposition mechanisms affect centromeric chromatin remains to be determined.

Several cancers display DAXX upregulation, with further upregulation occurring in metastases compared to primary tumors in breast, prostate and colon cancers (37). Deciphering the ATRX-independent roles of DAXX may hold the key to uncovering critical functions perturbed in DAXX-overexpressing cancers, as well as in cancers harboring *DAXX* mutations such as PanNETs (94,95) and pediatric high-grade gliomas (pHGGs) (36), while also providing novel therapeutic strategies for such cancers. In the case of pHGGs in particular, the epigenetic reprogramming afforded by driving mutations in histones H3.1/H3.3 and associated loss of function of the ATRX/DAXX complex offers an interesting background for combinatorial treatments that target genomic instability.

## Supplementary Material

gkad1141_supplemental_file

## Data Availability

The FACS analysis data (Fig. S2C) are available in Flow Repository (https://flowrepository.org/), and can be accessed with ID FR-FCM-Z5LR.

## Notes

Present address: Abhishek Bharadwaj Sharma, Department of Biochemistry, University of Oxford, Oxford OX1 3QU, UK.

Present address: Anthony J. Rizzo, Duke University, Department of Biomedical Engineering, Durham, NC 27708, USA.

Present address: Mindy K. Graham, Department of Urology, Northwestern University, Feinberg School of Medicine, Chicago, IL 60611, USA.
