# Supplementary material for: DAXX promotes centromeric stability independently of ATRX by preventing the accumulation of R-loop-induced DNA double-stranded breaks
Source: Nucleic Acids Res. 2023 Dec 1;52(3):1136–55. doi: 10.1093/nar/gkad1141 (PMC10853780; doi:10.1093/nar/gkad1141)
Supplement: gkad1141_supplemental_file [file gkad1141_supplemental_file.pdf]

Pinto et al. Supplementary Figures and Legends

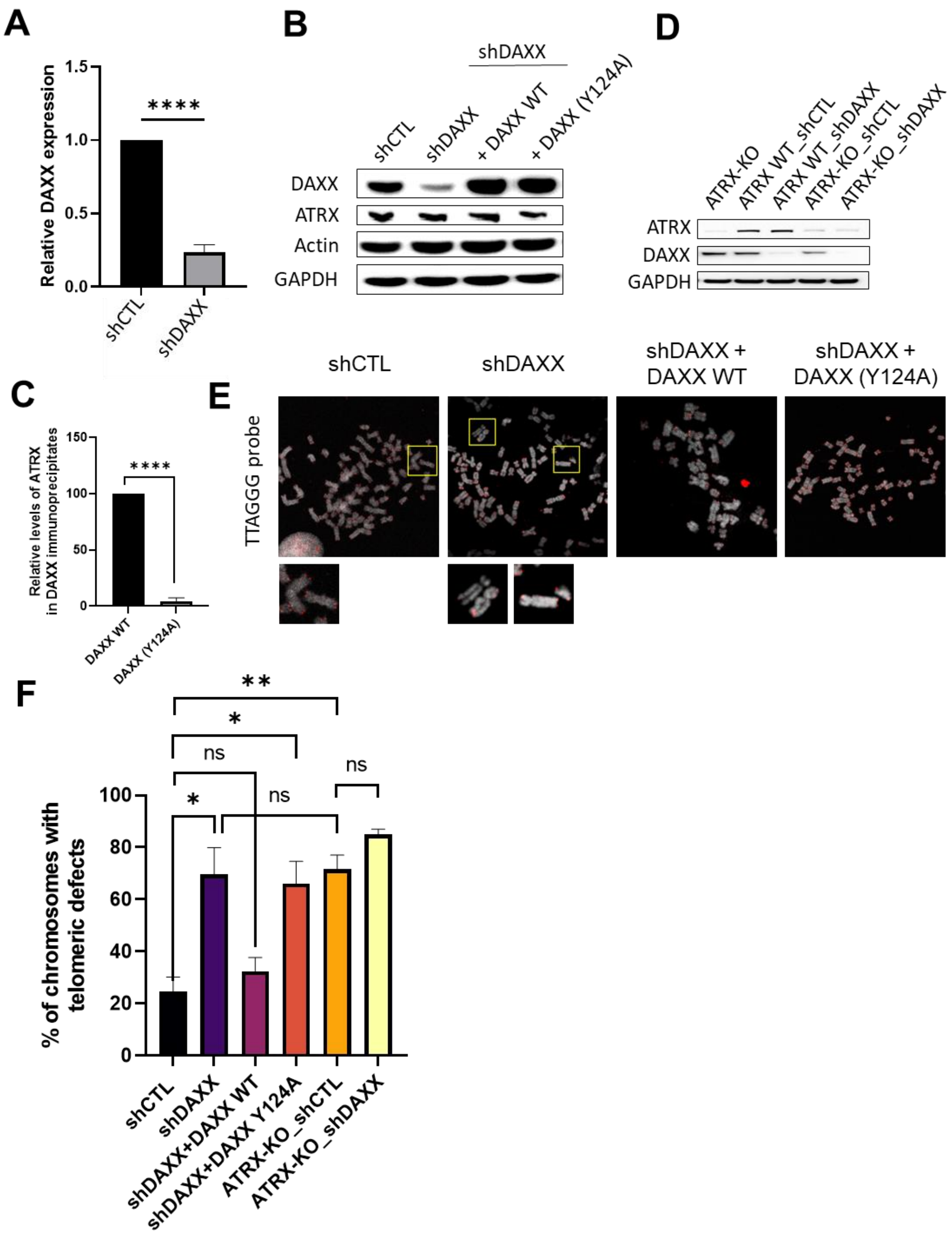
A
Relative DAXX expression
1.5
1.0
0.5
0.0
****
shCTL
shDAXX
B
shDAXX
shCTL
shDAXX
+ DAXX WT
+ DAXX (Y124A)
DAXX
ATRX
Actin
GAPDH
D
ATRX-KO
ATRX WT_shCTL
ATRX WT_shDAXX
ATRX-KO_shCTL
ATRX-KO_shDAXX
ATRX
DAXX
GAPDH
C
Relative levels of ATRX in DAXX immunoprecipitates
150
100
50
0
****
DAXX WT
DAXX (Y124A)
E
shCTL
shDAXX
shDAXX + DAXX WT
shDAXX + DAXX (Y124A)
TTAGGG probe
F
% of chromosomes with telomeric defects
100
80
60
40
20
0
**
*
ns
ns
*
ns
shCTL
shDAXX
shDAXX+DAXX WT
shDAXX+DAXX Y124A
ATRX-KO_shCTL
ATRX-KO_shDAXX

Figure S1. (**A**) Efficiency of *DAXX* depletion achieved by a shRNA targeting its 3'UTR compared to a control, non-silencing shRNA, as determined at the mRNA level by RT-qPCR. Statistical significance is reported as p-value < 0.0001****. Error bars represent the standard error of mean (s.e.m). (**B**) WB showing the levels of DAXX in control and DAXX-depleted cells, as well as in shDAXX cells ectopically expressing DAXX WT or DAXX (Y124A). Actin and GAPDH were used as loading controls. (**C**) Quantification of the relative levels of ATRX in immunoprecipitates of HA-tagged DAXX (Y124A)-expressing cells, compared to HA-tagged DAXX WT-expressing cells. The results are from 2 biological replicates. Statistical significance is reported as p-value < 0.0001 ****. The error bars represent the standard deviation (SD). (**D**) Immunoblot analysis of DAXX and ATRX in whole cell extracts of the indicated cell lines. A complete description of the CRISPR-generated ATRX-KO cell line is presented in Ref. 46. Notably, a faint band with an apparent electrophoretic mobility similar to that of ATRX is detected in ATRX-KO cells. The identity of this band is discussed in Materials and Methods. (**E**-**F**) Representative pictures of FISH on metaphase spreads with C3-TELO (TTAGGG) PNA probe (red), and related quantification of telomere defects per metaphase spread in the indicated cell lines (F). At least 15 spreads (per biological replicate) for each sample and condition were used for the quantification. Statistical significance is reported as: p-value < 0.05 *, p-value < 0.01 **, p-value > 0.05 ns. The error bars represent the s.e.m.

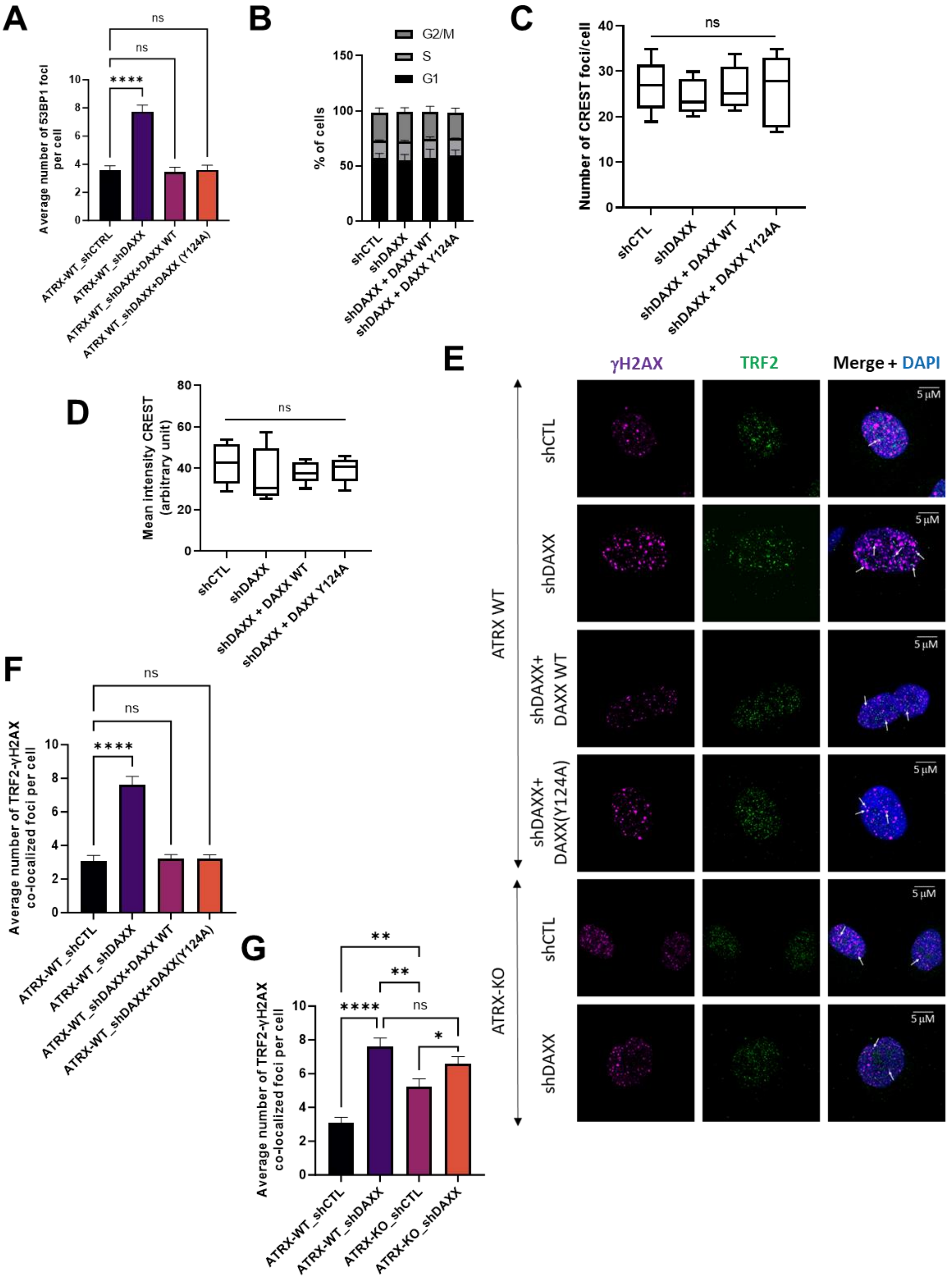
A
B
C
D
E
F
G
Average number of 53BP1 foci per cell
ATRX-WT_shCTRL
ATRX-WT_shDAXX
ATRX-WT_shDAXX+DAXX WT
ATRX WT_shDAXX+DAXX (Y124A)
G2/M
S
G1
% of cells
Number of CREST foci/cell
Mean intensity CREST (arbitrary unit)
shCTL
shDAXX
shDAXX + DAXX WT
shDAXX + DAXX Y124A
γH2AX
TRF2
Merge + DAPI
5 µM
ATRX WT
ATRX-KO
shDAXX+ DAXX WT
shDAXX+ DAXX(Y124A)
Average number of TRF2-γH2AX co-localized foci per cell
ATRX-WT_shCTL
ATRX-WT_shDAXX+DAXX(Y124A)
ATRX-KO_shCTL
ATRX-KO_shDAXX
ns
****
**
*

New Figure S2. (**A**) Quantification of 53BP1 foci in the indicated SF188 cell derivatives. Statistical significance is reported as p-value < 0.0001****, p-value > 0.05 ns. See Figure 2A for representative IF images. (**B**) Flow cytometry analysis. Shown are the percentage of the indicated SF188 cell derivatives in G1, S and G2/M based on propidium iodide staining (N = 3).The error bars represent the s.e.m. (**C**) Average number of CREST foci in SF188 cells (via indirect IF). (**D**) Mean intensity of CREST signal per cell in SF188 cells (via indirect IF). (**E**) Indirect IF analysis of γH2AX foci (magenta) and TRF2 foci (green) in the indicated cell lines. White arrows point to representative co-localizing γH2AX and TRF2 foci (TIFs). (**F-G**) Related quantification of TIFs in control and DAXX-depleted cells, as well as in DAXX-depleted cells expressing DAXX WT or DAXX (Y124A) (F) or in ATRX-KO and ATRX-KO_shDAXX cell lines (G). All experiments account for three biological replicates. At least 70 nuclei for each sample and condition were used per replicate for quantification. Statistical significance is reported as: p-value < 0.05 *, p-value < 0.01 **, p-value <0.0001 ****, p-value > 0.05 ns. Error bars represent the s.e.m. Scale bar: 5 μm.

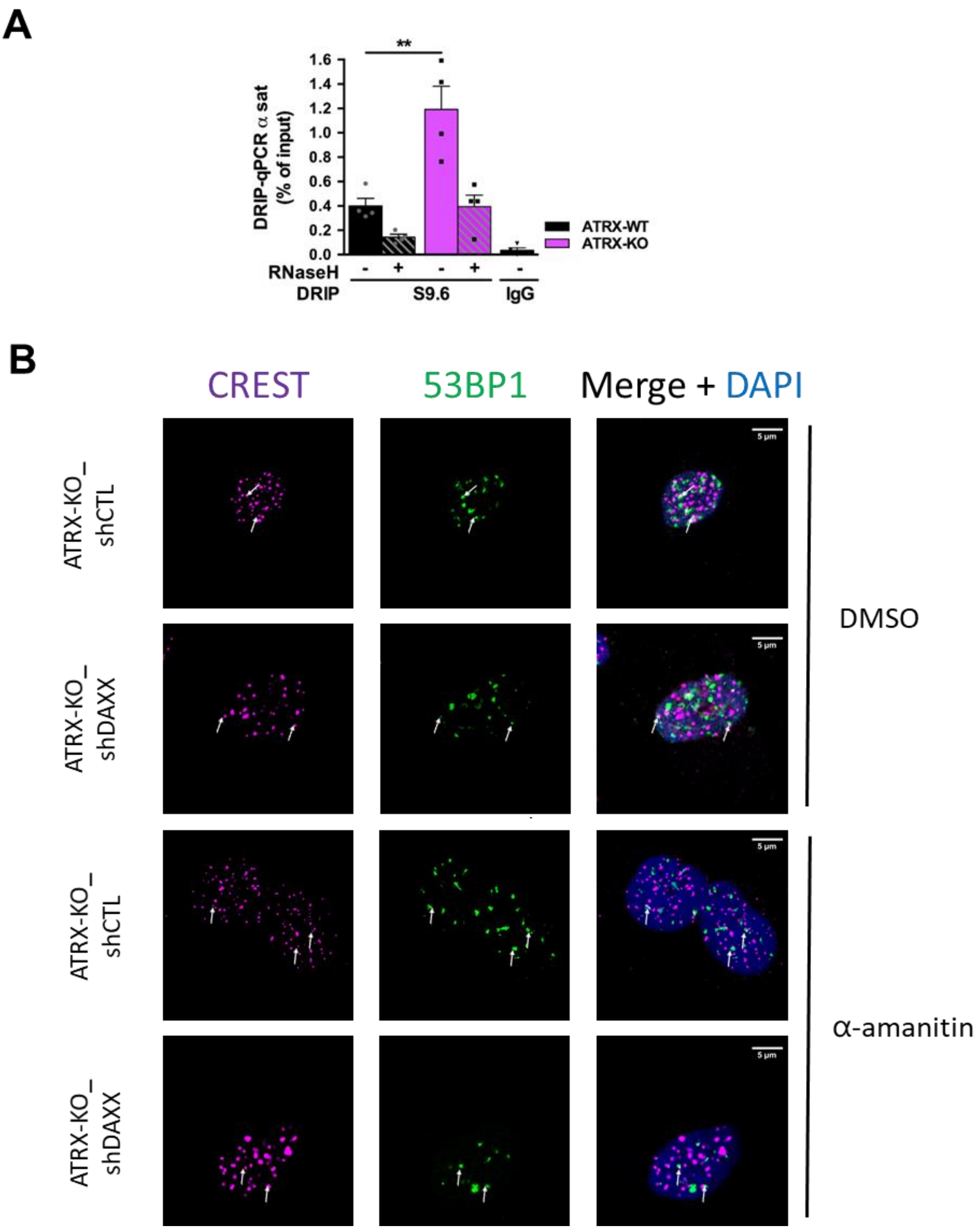

Figure S3. (**A**). DRIP-qPCR analysis at α-SAT repeats in ATRX-WT and ATRX-KO cells. RNase H treatment was performed on half of each sample prior to IP with the S9.6 antibody. Displayed is the R-loop enrichment (as percent input) as mean ± s.e.m. Experiments were carried out in biological quadruplicates. (**B**) Representative IF images of 53BP1 foci (green) and CREST foci (magenta) in the indicated ATRX-KO derivative cell lines left untreated or treated with α-amanitin (2.5 μM) for 16h. White arrows point to representative centromeric 53BP1 foci. See Figure 3F for related quantification. Scale bar: 5 μm. Statistical significance is reported as: p-value < 0.01 **.

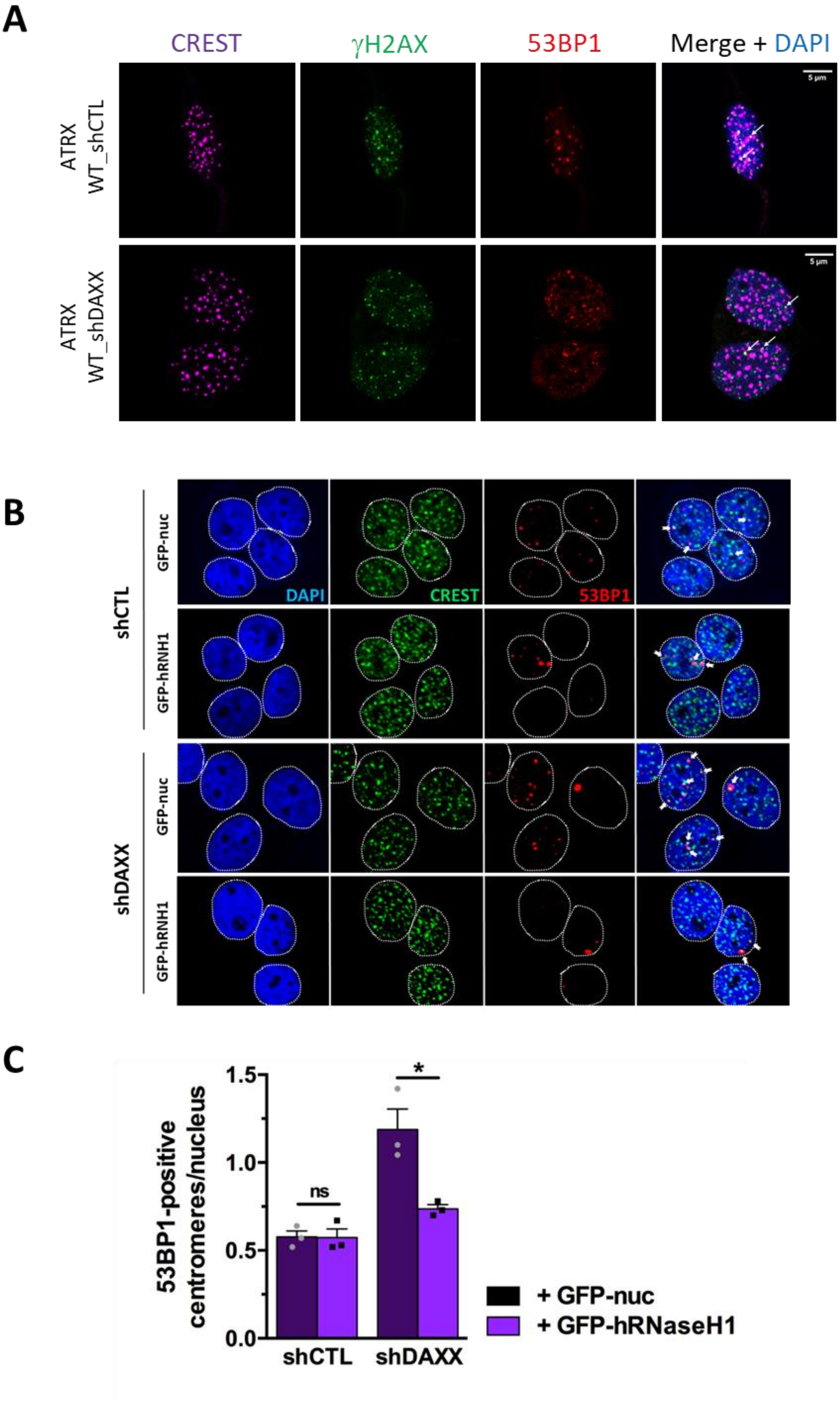
A
CREST
γH2AX
53BP1
Merge + DAPI
ATRX WT_shCTL
ATRX WT_shDAXX
5 μm
B
shCTL
shDAXX
GFP-nuc
GFP-hRNH1
DAPI
CREST
53BP1
C
53BP1-positive centromeres/nucleus
1.5
1.0
0.5
0.0
ns
*
shCTL
shDAXX
+ GFP-nuc
+ GFP-hRNaseH1

Figure S4. (**A**) Representative IF images of 53BP1 foci (red), γH2AX foci (green) and CREST foci (magenta) in control and DAXX-depleted cells stably expressing a hRNAseH1 from a lentiviral vector. See Figure 2A for IF images of untransduced cells. White arrows point to representative centromeric DSBs. See Figures 4D and 4E for related quantification. Scale bar: 5 μm. (**B-C**) Representative IF images of CREST foci (green) and 53BP1 foci (red) in control and DAXX-depleted cells transiently expressing GFP-hRNH1 or GFP-nuc (B) and related quantification (C). White arrows point to representative co-localizing 53BP1 and CREST foci. Note that the GFP signal is lost during the CSK pre-extraction step. Statistical significance is reported as: p-value < 0.05 *. The error bars represent the s.e.m.

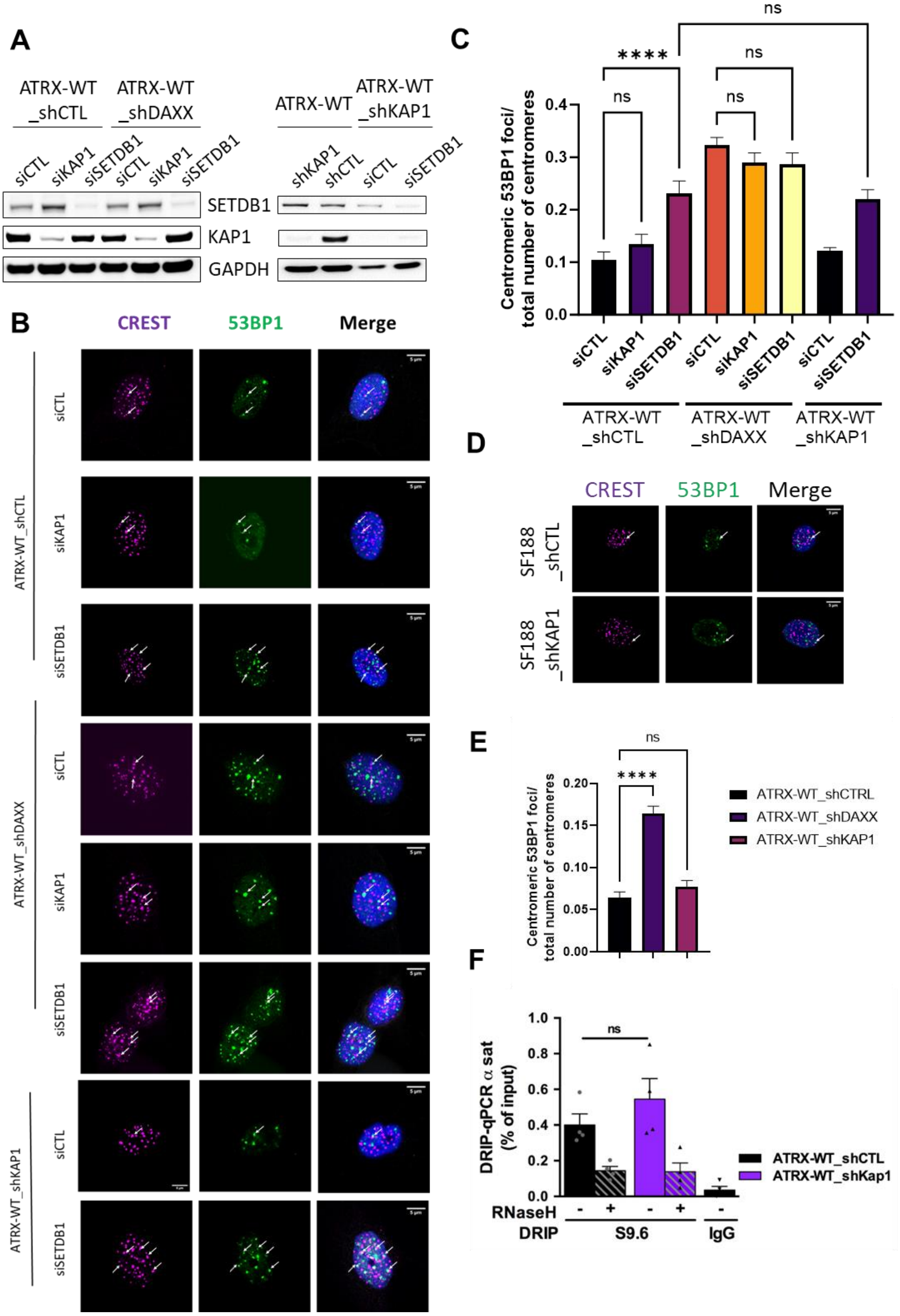
A
ATRX-WT _shCTL
ATRX-WT _shDAXX
ATRX-WT
ATRX-WT _shKAP1
siCTL
siKAP1
siSETDB1
shKAP1
shCTL
SETDB1
KAP1
GAPDH
B
CREST
53BP1
Merge
ATRX-WT_shCTL
ATRX-WT_shDAXX
ATRX-WT_shKAP1
C
Centromeric 53BP1 foci/ total number of centromeres
ns
****
D
SF188 _shCTL
SF188 _shKAP1
E
ATRX-WT_shCTRL
ATRX-WT_shDAXX
ATRX-WT_shKAP1
F
DRIP-qPCR α sat (% of input)
ATRX-WT_shCTL
ATRX-WT_shKap1
RNaseH
DRIP
S9.6
IgG

Figure S5. Epistatic relationships between DAXX, SETDB1 and KAP1 in the prevention of centromeric DNA damage. (**A**) Western blot analysis of SETDB1 and KAP1 in whole cell lysates of the indicated cells lines. Left panel: siRNA experiments; right panel: shKAP1, as well as siSETDB1 in shKAP1 experiments. GAPDH was used as loading control. (**B**) Representative IF images of CREST foci (magenta) and 53BP1 foci (green) in WT and DAXX-depleted SF188 cells transfected with control, non-silencing siRNAs (siCTL) or with siRNAs targeting SETDB1 (siSETDB1) or KAP1 (siKAP1). White arrows point to representative centromeric 53BP1 foci. (**C**) Related quantification of centromeric 53BP1 foci in the indicated cell lines. (**D-E**) Representative IF images of CREST foci (magenta) and 53BP1 foci (green) in WT and KAP1-depleted SF188 cells (D) and related quantification of centromeric 53BP1 foci (E). All experiments account for three biological replicates. At least 50 nuclei for each sample and condition were quantified for every biological replicate. Scale bar: 5 μm. (**F**) Loss of KAP1 does not elicit centromeric R-loop accumulation. DRIP-qPCR analysis at α-SAT repeats in the indicated SF188 cell derivatives. Displayed is the R-loop enrichment (as percent input) as mean ± s.e.m. Experiments were carried out in biological duplicates. Statistical significance is reported as: p-value < 0.0001 ****, p-value > 0.05 ns. All error bars represent the s.e.m.

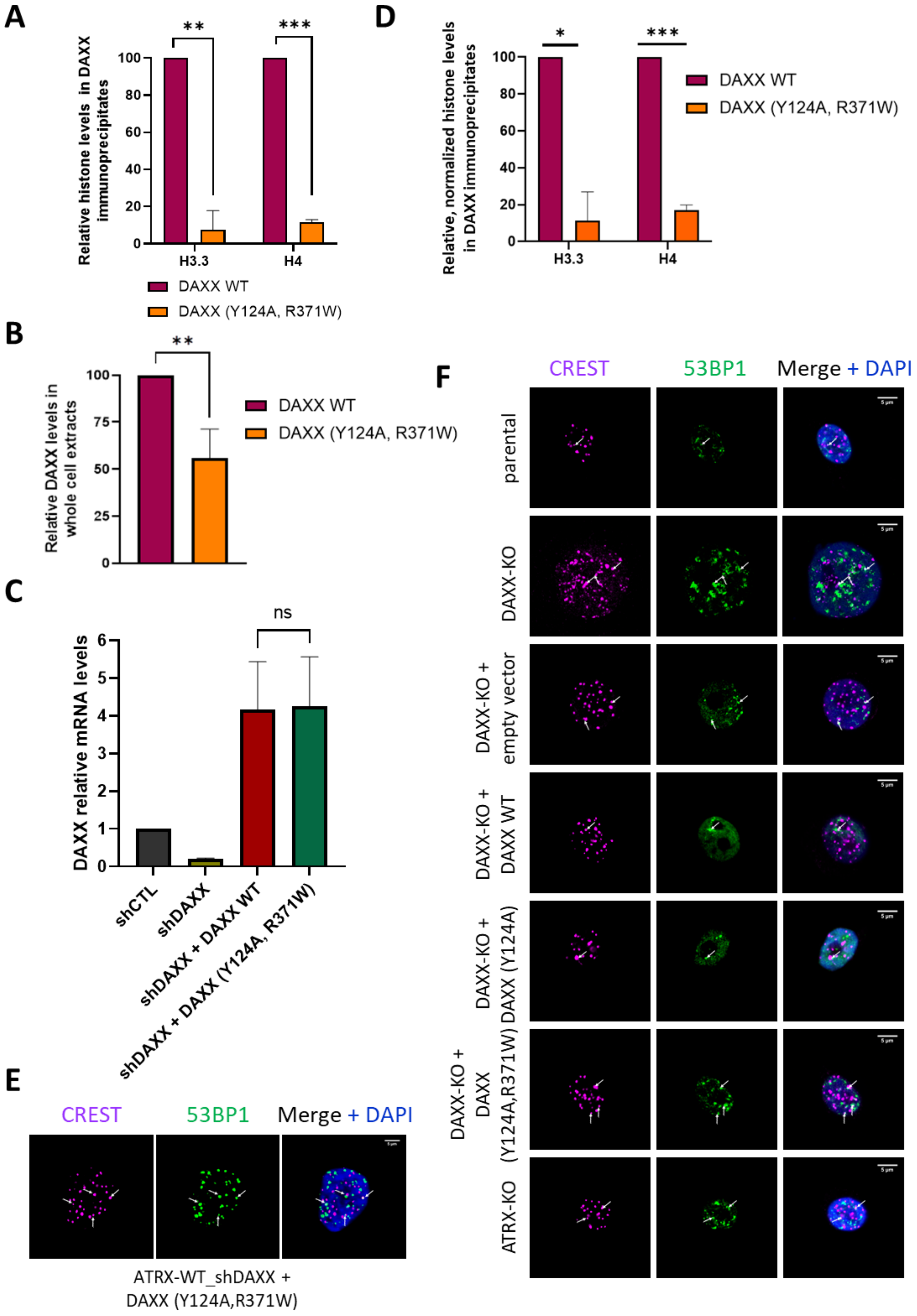
A
Relative histone levels in DAXX immunoprecipitates
**
***
H3.3
H4
DAXX WT
DAXX (Y124A, R371W)
B
Relative DAXX levels in whole cell extracts
**
DAXX WT
DAXX (Y124A, R371W)
C
DAXX relative mRNA levels
ns
shCTL
shDAXX
shDAXX + DAXX WT
shDAXX + DAXX (Y124A, R371W)
D
Relative, normalized histone levels in DAXX immunoprecipitates
*
***
H3.3
H4
DAXX WT
DAXX (Y124A, R371W)
E
CREST
53BP1
Merge + DAPI
ATRX-WT_shDAXX +
DAXX (Y124A,R371W)
F
CREST
53BP1
Merge + DAPI
parental
DAXX-KO
DAXX-KO + empty vector
DAXX-KO + DAXX WT
DAXX-KO + DAXX (Y124A)
DAXX-KO + DAXX (Y124A,R371W)
ATRX-KO

Figure S6. (**A**) Quantification of H3.3 and H4.4 levels in the indicated DAXX immunoprecipitates (N=2). (**B**) Relative amounts of DAXX (Y124A, R3371W) compared to WT DAXX in cell extracts expressing the indicated constructs (N=3). (**C**) RT-qPCR analysis of *DAXX* mRNA levels in control and DAXX-depleted cells, as well as in DAXX-depleted cells expressing WT DAXX or DAXX (Y124A, R3371W). *GAPDH* was used as housekeeping gene. The data were normalized to that of shCTL cells. (**D**) Quantification of H3.3 and H4.4 levels in the indicated DAXX immunoprecipitates, normalized to the levels of DAXX WT or DAXX (Y124A, R371) detected in the immunoprecipitates (N=2). The error bars represent the s.d. (panels A, B, D) or s.e.m (panel C). (**E**) Representative IF images of 53BP1 foci (green) and CREST foci (magenta) in DAXX-depleted SF188 cells expressing DAXX (Y124A, R371W). White arrows point to representative CREST- 53BP1 colocalized foci. See Figure 2A for representative images of control, as well as DAXX depleted cells and DAXX-depleted cells expressing the ATRX binding mutant DAXX (Y124A). See Figure 6B for related quantification. (**F**) Representative IF images of 53BP1 foci (green) and CREST foci (magenta) in the indicated BON-1 cell derivatives. White arrows point to representative centromeric 53BP1 foci. See Figure 6D for related quantification. Scale bar: 5 μm. Statistical significance is reported as: p-value < 0.01 **, p-value > 0.05 ns. The error bars represent the s.e.m.

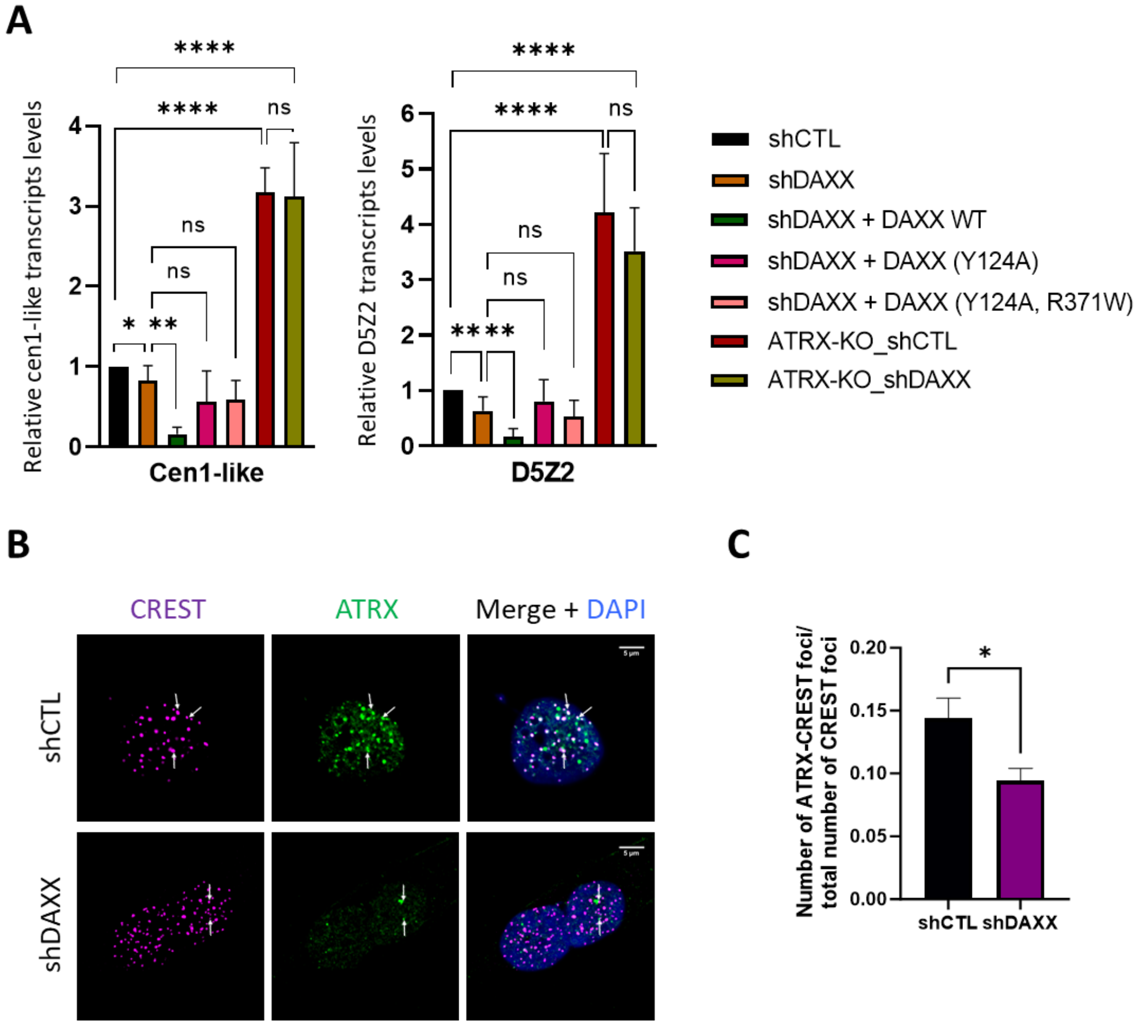

Figure S7. (**A**) Impact of DAXX and ATRX loss on alphoid DNA transcription. RT-qPCR analysis of centromeric RNA transcripts levels in the indicated cell lines. Data were normalized to GAPDH and compared to shCtrl and are shown as mean ± s.d. n≥3 independent experiments. Statistical significance (t-test) is reported as: p-value < 0.05 *, p-value < 0.01 **, p-value < 0.001 ***, p-value < 0.0001 ****, p-value > 0.05 ns. (**B-C**) Representative IF images of CREST foci (magenta) and ATRX foci (green) in shCTL and shDAXX cells (B), and related quantification of centromeric ATRX foci (C). The experiment accounts for three biological replicates with at least 40 nuclei studied per replicate. Statistical significance is reported as: p-value < 0.05 *. All error bars represent the s.e.m.
